# Programmable Spatiotemporal Magnetic Fields for Selective Multimodal Actuation and Deterministic Decoupling of Heterogeneous Magnetic Robots

**DOI:** 10.34133/research.1458

**Published:** 2026-10-14

**Authors:** Armando Ramos-Sebastian, Ja Sung Lee, Sung Hoon Kim

**Affiliations:** ^1^ Center for Advanced Photonic Materials, College of Science and Technology, Korea University, Sejong, Republic of Korea.; ^2^ Department of Electronics and Information Engineering, College of Science and Technology, Korea University, Sejong, Republic of Korea.; ^3^ Interdisciplinary Graduate Program for Artificial Intelligence Smart City Convergence Technology, Korea University, Sejong, Republic of Korea.

## Abstract

Magnetic milli/microrobots hold strong potential for biomedical applications, but their deployment as multi-robot systems is limited by short-range magnetic coupling, which prevents independent control in confined environments. Here, we present an expanded field-free region (FFR)-based control framework for spatially selective actuation of multiple magnetic torque-driven robots in confined environments. A generalized selective-control criterion incorporating the minimum rotating magnetic field required for robot actuation enables the framework to accommodate robots with different magnetic responses and operating conditions. Through programmable displacement and oscillation of the FFR, the framework extends conventional selective locomotion to include velocity regulation under a constant rotating magnetic field and oscillation-induced stick–slip locomotion. For strongly interacting permanent-magnet robots, a dedicated decoupling field reorients their magnetic moments to transition the inter-robot interaction from attraction to repulsion, enabling subsequent deterministic separation, whereas weakly interacting low-remanence robots can be separated using conventional FFR-based selective locomotion without dedicated decoupling. The framework is validated across different fluid viscosities and robots with distinct magnetic properties and is further integrated with robot redistribution and FFR-selective magnetic heating to achieve spatially regulated thermal responses. Finally, proof-of-concept demonstrations show clot-removal and targeted-delivery emulation and selective robot navigation in a 3-dimensional cerebrovascular phantom. These results establish an expanded FFR-based framework for sequential selective control of magnetic robots in confined environments and support its potential use in imaging-compatible robotic platforms for biomedical interventions.

## Introduction

Magnetic milli/microrobots have emerged as promising tools for biomedical applications. Recent advances in robot design and task-specific functionality have enabled applications such as vascular mapping [1] and embolization [2], extending beyond the capabilities of earlier generations of magnetic systems [3,4]. In vivo demonstrations in animal models, including intracranial tumor therapy and vascular navigation, further highlight the translational potential of these devices [5–9]. Despite this rapid progress, most current magnetic robotic systems still operate as either single agents or homogeneous swarms [10,11], limiting the flexibility and efficiency of multi-step therapeutic procedures. In particular, the inability to independently control multiple robots in close proximity remains a major obstacle to the deployment of magnetic multi-robot systems in confined, biomedically relevant environments.

Several strategies have been explored to achieve selective control of magnetic robots. One approach introduces heterogeneity among robots, such as differences in magnetization [12,13], geometry [14,15], or frequency-dependent actuation characteristics [16,17] so that individual robots exhibit distinct responses to the same global magnetic field generated by systems such as tri-axial Helmholtz coils [18,19]. Selectivity is then achieved by changing the global field parameters to selectively actuate a specific robot or robot subset. Although effective in specific cases, this strategy is difficult to scale because only a limited number of robots can be designed or fabricated with sufficiently distinct actuation responses within a practical field range. Moreover, simultaneous differential responses do not necessarily provide arbitrary simultaneous independent control, as the robots remain coupled through the same global magnetic input. Another approach uses local magnetic fields to spatially address identical robots [20–23]. This can potentially enable simultaneous independent control when sufficient independently controllable field sources are available; however, the required hardware generally increases with the number of independently controlled robots or regions, increasing system complexity, power consumption, and hardware burden. More importantly, these approaches do not explicitly address the loss of independent control caused by strong inter-robot magnetic coupling at short separation distances. In practice, this limitation has largely been avoided by maintaining sufficient inter-robot spacing rather than by actively restoring independent control after coupling occurs [24]. A comparison of representative selective-control strategies, including their selectivity mechanisms, control modes, advantages, and limitations, is provided in Table S1.

An alternative route to selective locomotion is based on spatial magnetic-field shaping. In particular, a selection field (SF) creates a field-free region (FFR), from which the magnetic field magnitude increases in all directions [25,26]. This principle is well established in magnetic particle imaging (MPI), where the FFR is used to spatially encode the magnetization response of magnetic nanoparticles (MNPs) under an alternating magnetic field (AMF) [27]. It has subsequently been adapted for robotic actuation, initially for the spatially selective control of helical milli-machines [28] and later for FFR-based selective locomotion of magnetic torque-driven robots in confined channels [25,29,30]. As summarized in Fig. 1A, when a rotating magnetic field (RMF) is uniformly applied throughout the workspace, robots that can follow the applied field undergo RMF-driven propulsion. In conventional FFR-based selective locomotion, however, only robots located near the FFR remain sufficiently responsive to the RMF to achieve sustained locomotion, whereas the increasing SF magnitude away from the FFR suppresses RMF-driven rotation and effectively immobilizes nontarget robots. This enables sequential spatial addressing without requiring robot-specific magnetic or geometric encoding. However, conventional FFR-based selective locomotion has not addressed the loss of independent control that occurs when strong inter-robot magnetic interactions lead to magnetic coupling at short separation distances. Given the increasing interest in MPI-compatible robotic actuation and spatially selective magnetic heating [26,31–33], FFR-based control provides a particularly attractive strategy for selective magnetic robotic operation in confined and tubular biomedical environments.

**Fig. 1.**
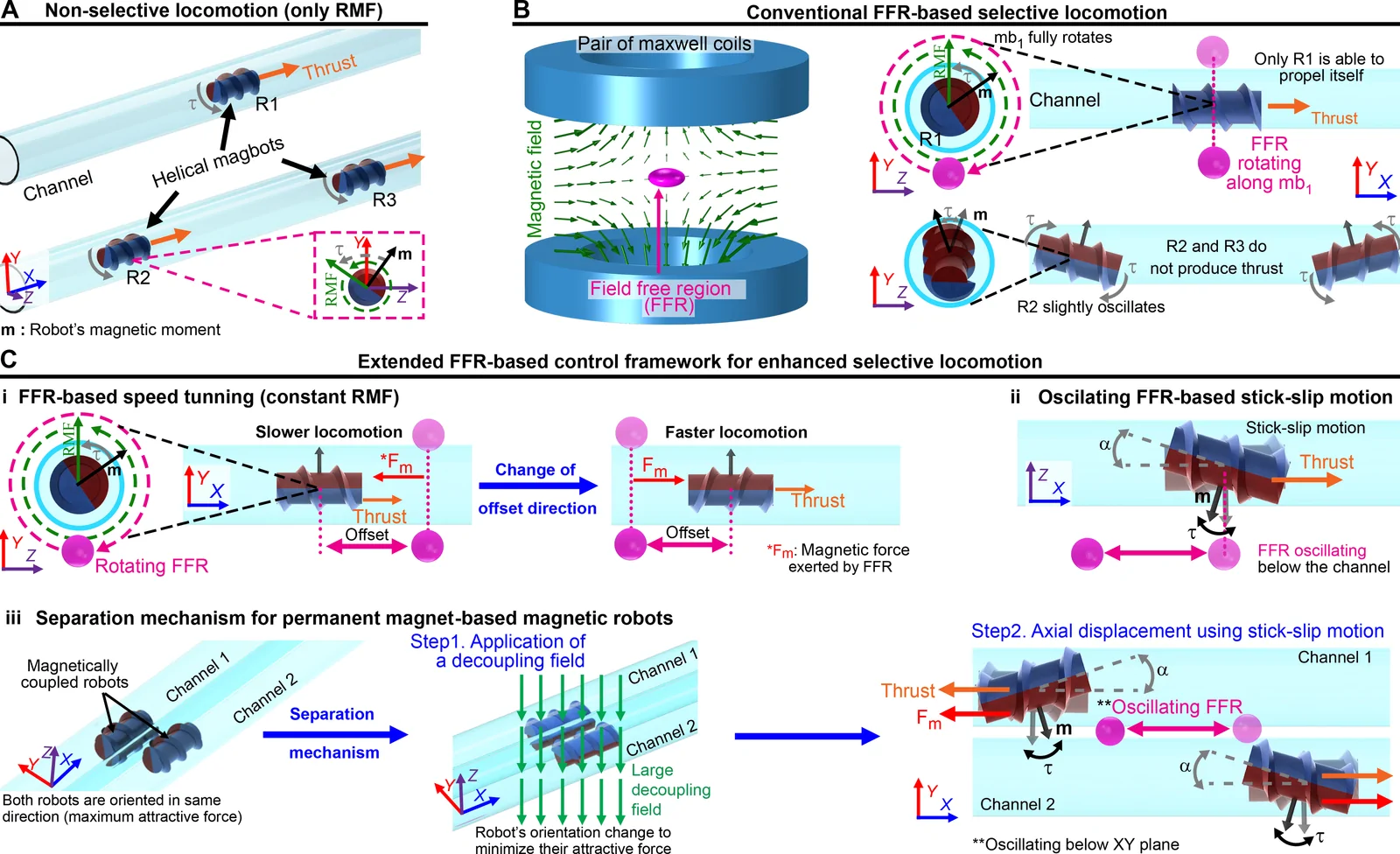
Extended FFR-based control framework for selective locomotion, dynamic tuning, and decoupling of channel-confined magnetic robots. (A) Under a uniformly applied RMF, all magnetic robots (R1 to R3) experience a magnetic torque (τ) that causes them to rotate and propel along their respective channels. (B) Conventional FFR-based selective locomotion relies on an FFR, generally generated by a pair of Maxwell coils, rotating through the selected robot R1. This allows R1 to fully rotate and propel, while the other robots remain locked in position. (C) Our extended FFR-based framework introduces 3 mechanisms: (i) speed tuning, (ii) stick–slip locomotion, and (iii) separation of magnetically coupled robots. (i) Speed tuning is achieved by offsetting the FFR from the selected robot opposite to or along the intended locomotion direction such that the resulting magnetic force (F_m_) decreases or increases the net propulsive force, respectively. (ii) Stick–slip locomotion is achieved by oscillating the FFR opposite to the desired locomotion direction, inducing periodic robot oscillations (by an angle α) and interactions between the robot ends and the channel walls that produce net propulsion. (iii) Separation is achieved in 2 steps: first, a sufficiently strong decoupling field is applied to reorient the coupled robots and reduce their mutual magnetic attraction; subsequently, the FFR is oscillated between the robots, causing them to propel in opposite directions and separate.

However, current FFR-based robotic control remains limited in several respects. Previous studies have established FFR-based selective locomotion by positioning an FFR at a target robot while suppressing the response of nontarget robots. Nevertheless, the corresponding selection conditions have been primarily developed for hard-magnetic robots and do not explicitly account for the minimum field magnitude required to actuate robots with different magnetic responses [29]. Moreover, conventional FFR-based approaches have largely treated the FFR as a means of selecting which robot is actuated, rather than exploiting its spatial and temporal modulation to regulate robot dynamics. Most importantly, existing selective-control strategies generally assume that the robots remain sufficiently separated to preserve independent actuation. At short inter-robot distances, magnetic interactions can instead cause robots to couple, at which point conventional FFR-based selection alone can no longer independently control them.

Here, we develop an expanded FFR-based control framework that extends conventional selective locomotion both before and after magnetic coupling. Before coupling, the framework generalizes the selective-control condition by incorporating the minimum RMF magnitude (B_RMFmin_) required for robot actuation, enabling the same selection criterion to be applied to robots with substantially different magnetic responses. Beyond conventional FFR-based selection, deliberate spatial displacement of the FFR is used to regulate robot velocity under a constant RMF, while temporal oscillation of the FFR generates directional stick–slip locomotion in confined channels. After short-range magnetic coupling occurs, we introduce an active decoupling strategy in which a uniform decoupling field reorients the coupled magnetic moments from an attractive toward a repulsive interaction regime, followed by oscillating-FFR locomotion to physically separate the robots and restore independent control. Thus, rather than requiring robots to remain magnetically isolated, the proposed framework provides a mechanism for recovering selective controllability after undesired magnetic coupling.

In this work, programmable spatiotemporal magnetic fields refer to the deliberate spatial and temporal manipulation of the applied magnetic field distribution—including the position and motion of the FFR and the superposition of uniform magnetic fields—to determine where, when, and how individual magnetic robots respond within a shared workspace. Consistent with conventional FFR-based selective locomotion, the present framework is primarily developed for robots operating in confined environments, where physical boundaries constrain undesired SF-induced translational motion. The overall framework is summarized in Fig. 1, which distinguishes conventional FFR-based selective locomotion (Fig. 1B) from the control mechanisms introduced here: FFR-position-dependent velocity regulation (Fig. 1C-i), oscillation-induced stick–slip locomotion (Fig. 1C-ii), and deterministic decoupling of magnetically coupled robots (Fig. 1C-iii).

As illustrated in Fig. 1B, conventional FFR-based selective locomotion maintains the FFR centered on the target robot while rotating it around the robot under an applied RMF. In contrast, our extended framework deliberately manipulates the position and motion of the FFR relative to the target robot to achieve additional control functionalities (Fig. 1C). A small FFR offset along the locomotion axis can assist or oppose the robot motion, enabling fine velocity regulation under a constant RMF (Fig. 1C-i). At larger offsets, temporal oscillation of the FFR induces directional stick–slip locomotion in confined environments (Fig. 1C-ii). Finally, for magnetically coupled permanent-magnet robots (PMRs), a uniform decoupling field ($B_{D}$) is superimposed to reorient their magnetic moments beyond the attraction-to-repulsion transition, while the oscillating FFR subsequently drives their physical separation (Fig. 1C-iii). Together, these field-manipulation strategies extend the role of the FFR beyond conventional robot selection, enabling regulation of robot dynamics and recovery of independent control after magnetic coupling.

To systematically establish and evaluate these control mechanisms, we initially use PMRs as a model system. Building on the selective field ratio (*λ*) introduced in previous FFR-based studies, we extend the selection criterion by incorporating B_RMFmin_. This modification accounts for changes in the actuation threshold under different environmental conditions, including fluid viscosity, thereby extending the applicability of the criterion beyond the conditions considered previously. Using PMRs, we then experimentally demonstrate FFR-position-dependent velocity regulation, oscillation-induced stick–slip locomotion, and deterministic decoupling of magnetically coupled robots. The relationship between inter-robot distance and the decoupling field required to transition the coupled PMRs from attractive to repulsive interaction is further quantitatively evaluated and experimentally validated.

After establishing these mechanisms with PMRs, we investigate whether the selective-control criterion can be generalized to magnetic hydrogel robots with substantially different magnetic responses, including Fe_3_O_4_-based soft-magnetic robots (SMRs) and remanently magnetized NdFeB-containing hydrogel robots. Finally, we demonstrate the applicability of the framework to increasingly complex multi-robot scenarios, including sequential separation of magnetically coupled PMRs across 500-μm barriers (a dimension comparable to the thickness of some vascular walls [34]), selective locomotion in open 2-dimensional (2D) workspaces and a 3D cerebrovascular phantom, and multimodal functions involving spatially selective magnetic heating under continuous AMF exposure, clot-drilling, and targeted-delivery emulation using an electromagnetic system with specifications comparable to commercial preclinical MPI setups [35,36]. Collectively, these results extend FFR-based selective actuation beyond conventional selection among independently controllable robots toward a broader framework that regulates robot dynamics, accommodates different magnetic responses and environmental conditions, and restores independent control following short-range magnetic coupling.

## Results

### Field configuration for conventional FFR-based selective locomotion

As illustrated in Fig. 2, conventional FFR-based selective locomotion of magnetic robots requires 3 magnetic field components: an SF, an RMF, and a quasi-static magnetic field (SMF). As shown in Fig. 2A, in our system, the SF is generated by a pair of Maxwell coils whose axis coincides with the system’s reference *Z* axis and is defined as:

$$\mathrm{SF}=\mathrm{Gp}=\left[\begin{array}{ccc} -G_{z}/2 & 0 & 0\\ 0 & -G_{z}/2 & 0\\ 0 & 0 & G_{z} \end{array}\right]\left[\begin{array}{c} p_{x}\\ p_{y}\\ p_{z} \end{array}\right]$$ (1)

where ***G*** is the gradient tensor, ***p*** is the spatial position, and *G_z_* is the gradient magnitude along the *Z* direction. The radial gradient *G_r_* is the term *–G_z_*/2 of the tensor **G**. The FFR is located at the center of the coils, $p=\left[0,\;0,\;0\right]$, which defines the origin of the reference frame.

**Fig. 2.**
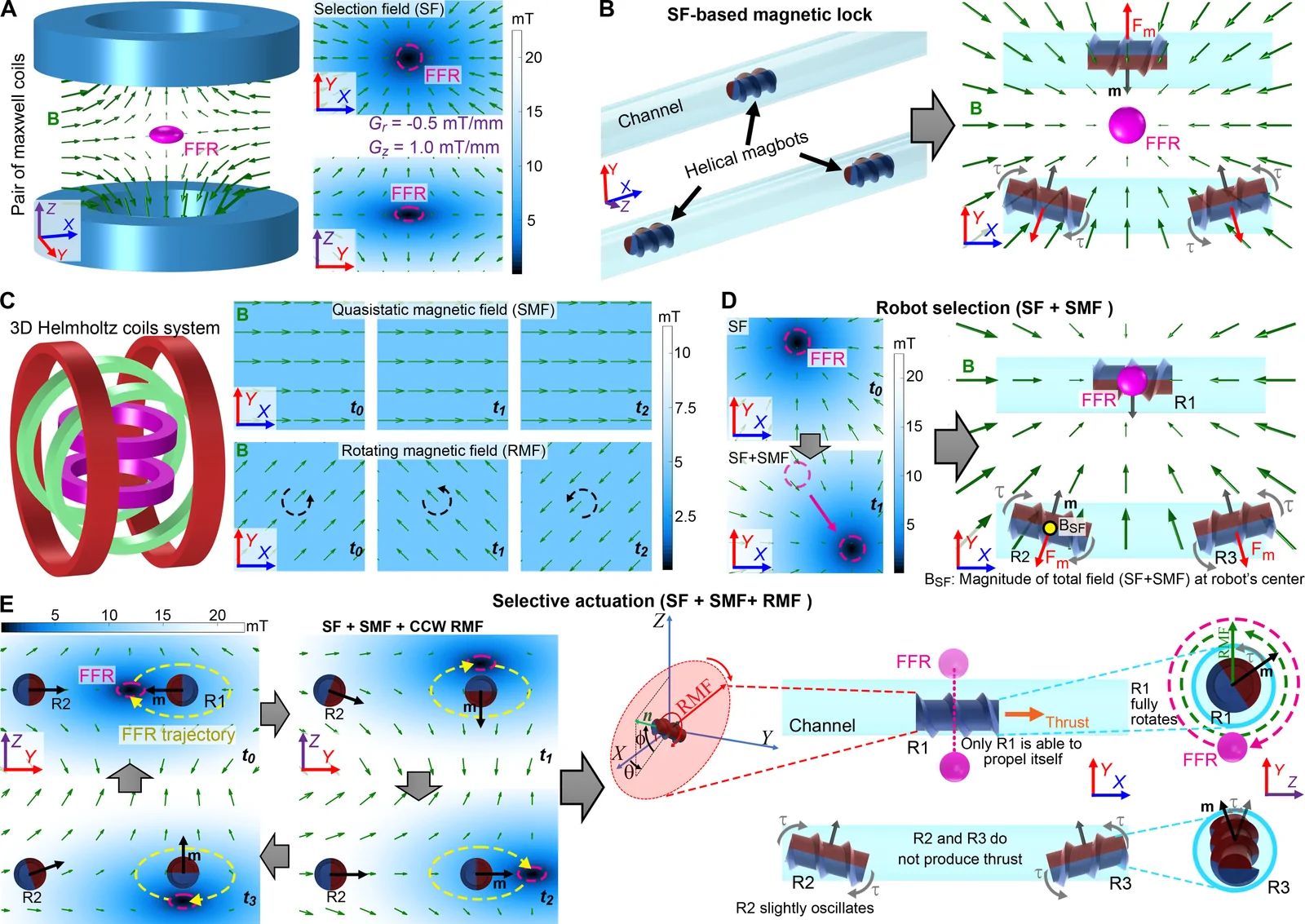
Conventional FFR-based selective locomotion of channel-confined magnetic robots. (A) A pair of Maxwell coils generates an SF, characterized by a FFR at its center. The magnetic field gradient along the axial direction (*G_z_*) is twice that along the radial direction (*G_r_*). (B) The gradient distribution of the SF exerts magnetic forces (F_m_) and torques (τ) that constrain the motion of robots confined within the channels. (C) A 3D Helmholtz coil system generates 2 types of spatially uniform magnetic fields: a quasi-SMF, which varies slowly, and an RMF. (D) For selective locomotion, an SMF is applied to shift the FFR from its initial position to the position of the target robot (R1), where it ideally experiences zero F_m_ and τ. (E) A CCW RMF, with its rotation axis defined by the azimuthal and polar angles *θ* and *φ*, is subsequently applied with the rotation axis aligned along the X axis, causing the FFR to rotate clockwise around the target robot. Because the theoretical SF magnitude at the FFR center is zero, R1 is primarily subjected to the RMF, allowing it to fully rotate and propel along direction n, which coincides with the RMF rotation axis. In contrast, the nontarget robots (e.g., R2), located outside the FFR, undergo only small oscillations and consequently exhibit negligible displacement.

The Maxwell coil pair has a mean diameter of 14 cm and consists of 200 turns, with *G_z_* given by:

$$G_{z}=k_{m}\;I_{m}$$ (2)

where *k_m_* is a constant related to geometrical properties of the coil pair and *I_m_* is the applied electric current. *k_m_* is equal to 35 × 10^−3^ T·m^−1^·A^−1^. As shown in Fig. 2B, applying an SF to a workspace containing channel-confined magnetic robots generates a magnetic torque (τ) that tends to align the robots with the FFR, as well as magnetic forces (F_m_) that push the robots against the channel boundaries. The resulting robot–wall interactions generate frictional forces that constrain the robots and lock their positions within the channels.

As shown in Fig. 2C, the SMF and RMF are generated by 3 orthogonal pairs of Helmholtz coils aligned along the *Z*, *X*, and *Y* axes, respectively. The corresponding coil pairs have mean diameters of 28, 44, and 60 cm and consist of 583, 430, and 602 turns, respectively. The resulting magnetic field is controlled as follows:

$$B={\left[k_{x}\;I_{x}\;k_{y}\;I_{y}\;k_{z}\;I_{z}\right]}^{T}$$ (3)

where *k* denotes constants related to the coils’ geometries (e.g., diameter and number of turns) and *I* represents the applied electric currents. The subscripts *x*, *y*, and *z* indicate the corresponding coil pairs aligned along each axis. The constants *k_x_*, *k_y_*, and *k_z_* are 1.76, 1.8, and 3.74 mT·A^−1^, respectively.

In this work, the workspace (50 mm × 50 mm × 40 mm) is located at the center of the electromagnetic coil system, which coincides with the origin of the coordinate system (***p*** = [0, 0, 0]). All channels and experimental containers used in this study are positioned within this central workspace. Because the Maxwell and Helmholtz coil pairs are substantially larger than the workspace, the generated SMF and RMF are considered spatially uniform within the workspace, while the SF is adequately described by Eq. (1). Accordingly, the robot and FFR positions are defined relative to the coordinate-system origin, independently of the specific channel geometry or its placement within the workspace.

As illustrated in Fig. 2D, the SMF—an offset field that changes only when repositioning the FFR—is used to match the steady-state position of the FFR with that of the target robot (R1 in the illustration). To position the FFR within the workspace, the required SMF is calculated as:

$$\mathrm{SMF}=-{\mathrm{Gp}}_{\mathrm{FFR}}$$ (4)

where $p_{\mathrm{FFR}}$ denotes the desired FFR position. When the FFR is positioned at the center of the target robot (e.g., R1), the robot ideally experiences neither τ nor Fm. In contrast, all nontarget robots remain magnetically locked by the SF. The magnetic field magnitude (B_SF_) at the center of the *i*th nontarget robot is given by:

$$B_{\mathrm{SF}}=\left\|G\left(p_{\mathrm{ri}}-p_{\mathrm{FFR}}\right)\right\|$$ (5)

where *i* denotes the index of the nontarget robot (e.g., R2) and $p_{\mathrm{ri}}$ denotes its spatial position.

The RMF, responsible for robot rotation, is given by:

$$\mathrm{RMF}=B_{\mathrm{RMF}}\left[\begin{array}{c} \text{cos}\;\theta \;\text{sin}\;\phi \;\text{sin}\;\omega t\;-\;\text{sin}\;\theta \;\text{cos}\;\phi \\ \text{cos}\;\theta \;\text{cos}\;\omega t+\text{sin}\;\theta \;\text{sin}\;\phi \;\text{sin}\;\omega t\\ -\text{cos}\;\phi \;\text{sin}\;\omega t \end{array}\right]$$ (6)

where *θ* and *φ* are the azimuthal and polar angles defining the rotation axis (aligned with the *X* axis when *θ* = *φ* = 0), *ω* is the angular frequency, and B_RMF_ is the field magnitude. As illustrated in Fig. 2E, after centering the FFR at R1, applying a counterclockwise (CCW) RMF perpendicular to the *X* axis causes the FFR to rotate in the opposite direction (clockwise) around R1. Meanwhile, R1 rotates following the RMF and propels itself along the *X* axis. As shown in the time-lapse sequence of the magnetic field distribution, nontarget robots, such as R2, experience only slight oscillations because their local magnetic field direction varies only slightly, resulting in negligible propulsion.

### Oscillating-FFR control for stick–slip motion

Figure 3A illustrates the procedure for FFR-based stick–slip locomotion. First, an arbitrary B_SF_, sufficiently large to align the robot magnetic moment **m** with the local SF, is defined. Subsequently, the initial FFR position $p_{F0}$ is calculated with an offset *r* perpendicular to the intended locomotion direction $\vec{n}$ as:

$$p_{\mathrm{FFR}}=p_{\mathrm{ri}}-\frac{B_{\mathrm{SF}}}{\left\|{\mathrm{Gn}}_{⊥}\right\|}\cdot n_{⊥}$$ (7)

where $n_{⊥}$ is an arbitrary unit vector perpendicular to $\vec{n}$. At this position, the magnetic force exerted by the SF pushes the robot against the channel wall opposite the FFR.

**Fig. 3.**
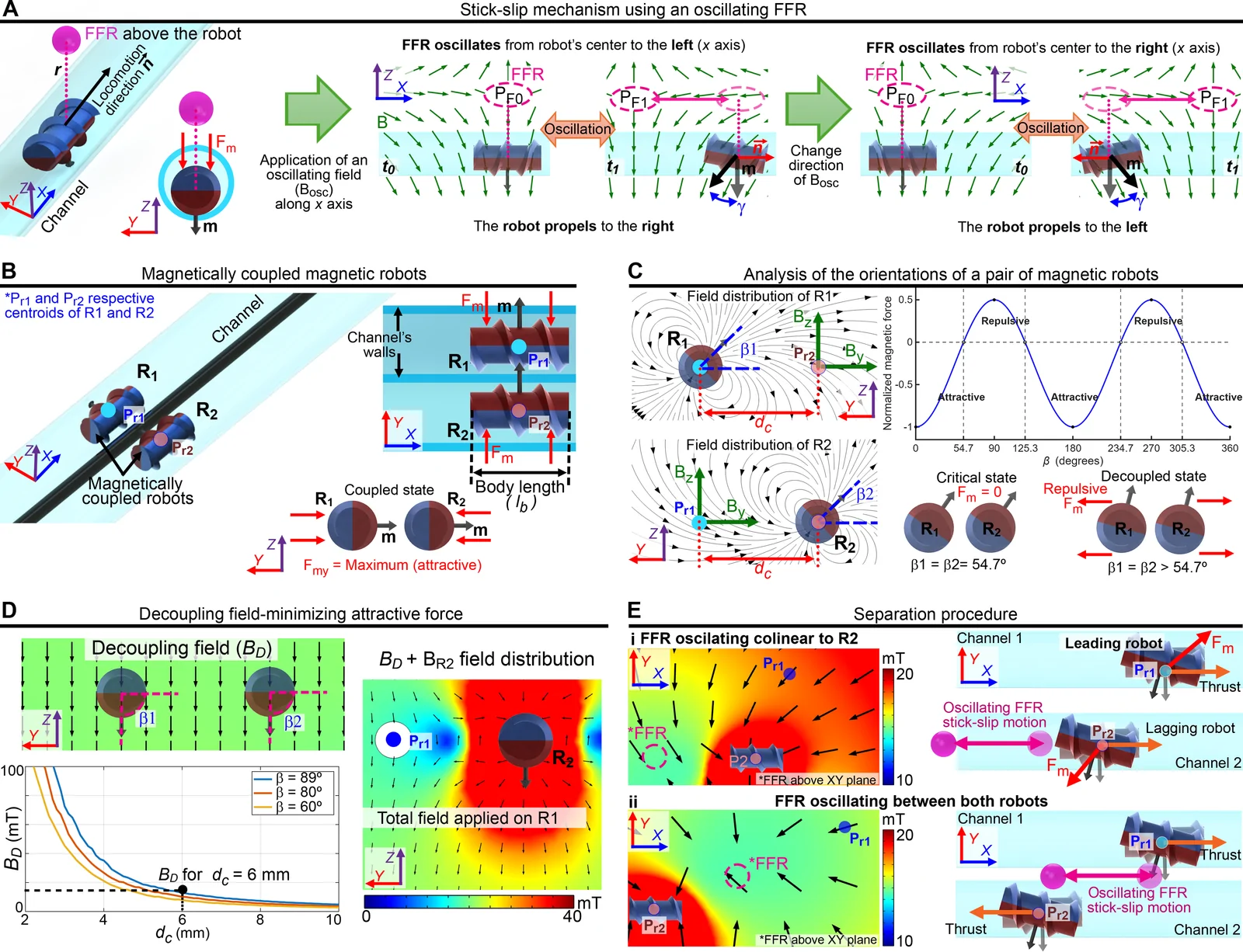
FFR-based stick–slip locomotion and separation mechanisms. (A) For stick–slip locomotion, the FFR is positioned with an offset (*r*) normal to the robot locomotion direction, $\vec{n}$. Subsequently, an oscillating magnetic field (B_osc_) is applied, causing the FFR to oscillate from the robot axis in the direction opposite to $\vec{n}$. (B) Two robots (R1 and R2) traveling through adjacent channels with a small separation distance become magnetically coupled, with their magnetic moments (*m*) aligned. Their attractive magnetic force (F_m_) is maximum along this configuration. (C) R1 generates a magnetic field distribution (B = [B_x_, B_y_, B_z_]) that exerts magnetic force and torque on R2, with centroid P_r2_, and vice versa. The magnitude and direction of the magnetic interaction at P_r1_ and P_r2_ depend on the orientations of R1 and R2 and their center-to-center separation distance, *d_c_*. Here, β = 0° corresponds to the initial maximally attractive configuration, in which the magnetic moments are collinear with the center-to-center axis. As both robots are reoriented by the same angle (β1 = β2 = β), their attractive interaction progressively decreases, reaching the theoretical critical angle β_c_ = 54.7°, at which the center-to-center force becomes zero under the ideal dipole approximation. Beyond this angle, the interaction becomes repulsive until the second critical angle at 125.3°, after which it becomes attractive again. (D) A uniform decoupling magnetic field (B_D_) is applied perpendicular to the initial magnetic moments of the coupled robots to reorient them beyond the critical angle β_c_, thereby transitioning their magnetic interaction from attractive to repulsive. The graph shows the estimated magnitude of B_D_ (*b_d_*) required to reorient R1 and R2 to β = 60°, 80°, and 90° at *d_c_* = 6 mm. (E) To complete the separation, FFR-based stick–slip locomotion is initially applied by oscillating the FFR next to R2, forcing the orientations of both robots beyond β_c_ and inducing a repulsive interaction. This interaction hinders the propulsion of R2, resulting in faster propulsion of R1 relative to R2. Once the robots are separated by at least half a body length (*l_b_*), the FFR is repositioned between the 2 robots and oscillated to induce stick–slip locomotion in opposite directions, further increasing their separation distance.

The FFR is then displaced from $p_{F0}$ to $p_{F1}$ in the direction opposite to $\vec{n}$. As the FFR is displaced, the resulting magnetic torque rotates the robot by an angle γ. Consequently, the end of the robot closer to $p_{F1}$ moves toward the FFR-facing channel wall, while the opposite end remains in contact with the opposite channel wall. The position $p_{F1}$ required to produce a rotation γ is calculated as:

$$p_{F1}=p_{F0}-r\text{tan}\left(\gamma \right)\vec{n}$$ (8)

For the robot to propel itself through stick–slip locomotion, it must periodically rotate from its initial orientation by an angle γ and return to its original orientation. Accordingly, the FFR is periodically displaced between $p_{F0}$ and $p_{F1}$. For this purpose, an oscillating magnetic field **B**_osc_, also generated by the Helmholtz coils, is applied and calculated as:

$$B_{\mathrm{osc}}=\frac{b_{\mathrm{osc}}\;\text{cos}\left(2\pi \mathrm{ft}\right)}{2}+\frac{b_{\mathrm{osc}}}{2}$$ (9)

where *b_osc_* is the required field magnitude ($b_{\mathrm{osc}}=r\text{tan}\left(\gamma \right)G\vec{n}$). The second term in Eq. (9) is constant and can therefore be incorporated into the SMF, simplifying the current control of the Helmholtz coils.

Figure 3A illustrates a simplified case of this control mechanism. For a robot with an intended locomotion direction along the +*X* axis, the FFR is initially positioned with an offset *r* from the robot axis along the *Z* axis. Oscillating the FFR from this position toward the −*X* direction induces stick–slip locomotion along the +*X* direction. Conversely, when the FFR oscillates toward the +*X* direction, the robot propels along the −*X* direction.

### Working principle for decoupling field and separation of magnetic robots

As illustrated in Fig. 3B, when 2 PMRs (e.g., R1 and R2) transit through closely spaced channels, their mutual magnetic interaction can become sufficiently strong to align their magnetic moments (**m**) and magnetically couple the robots, hindering their independent response to externally applied fields. Here, β_1_ = β_2_ = 0° is defined as the initial coupled configuration, in which the magnetic moments are collinear with the center-to-center axis and the attractive interaction is maximum. As illustrated in Fig. 3C, reorienting both robots changes their magnetic interaction. Considering the robots as ideal magnetic dipoles that are simultaneously reoriented by the same angle (β_1_ = β_2_ = β), the attractive force progressively decreases and reaches zero at the critical angle β_c_ = 54.7°. Beyond this angle, the interaction becomes repulsive, reaching maximum repulsion at β = 90°. Further rotation decreases the repulsive interaction until a second critical angle of β = 125.3°, beyond which the interaction becomes attractive again. This theoretical relationship provides the basis for selecting a robot orientation beyond β_c_ to facilitate magnetic decoupling.

In practice, the magnitude of the magnetic interaction also depends strongly on the magnetic field generated by each robot at the position of the neighboring robot and, consequently, on their center-to-center separation distance, *d_c_*. Because the robots are confined within separate channels, their collective rotation as a single magnetically coupled body is mechanically restricted, allowing their individual orientations to be manipulated by an externally applied field. Accordingly, as illustrated in Fig. 3D, a uniform decoupling field (B_D_) is applied perpendicular to their initial magnetic moments to reorient both robots beyond the theoretical critical angle β_c_, thereby transitioning their interaction from attractive to repulsive.

Determining the required B_D_ is nontrivial because the magnetic field generated by each robot at the position of the other changes continuously as their orientations change. Therefore, the spatial magnetic field distribution of each PMR was first obtained through finite-element simulations in COMSOL. The simulated field data were imported into MATLAB and interpolated to estimate the magnetic field generated by each robot at the centroid of the neighboring robot for their instantaneous positions and orientations. B_D_, applied perpendicular to the initial magnetization axes, was then iteratively increased from 100 μT in increments of 100 μT. At each iteration, the local total magnetic field at each robot was determined from the superposition of B_D_ and the magnetic field generated by the neighboring robot, and the equilibrium robot orientations were updated assuming that their magnetic moments align with the local total field. The procedure was repeated until both robots reached the prescribed orientation β. As shown in the corresponding graph in Fig. 3D, for *d_c_* = 6 mm, the calculated B_D_ values required to reach β = 60°, 80°, and 89° were 7.93, 10.13, and 13.22 mT, respectively. Since β = 60° is already beyond the theoretical critical angle of $54.7^{\circ }$, it represents a practical target orientation for transitioning the interaction from attractive to repulsive.

Once the magnitude of B_D_ (*b_d_*) is determined, its orientation within the system coordinate frame is obtained as follows:

$$B_{D}=b_{d}\left(\vec{n}\times {\vec{m}}_{0}\right)$$ (10)

where $\vec{n}$ and ${\vec{m}}_{0}$ denote the intended locomotion direction and the initial magnetic-moment orientation of the target robot (R1) when magnetically coupled to R2, respectively.

Once the attractive force is minimized, the separation procedure illustrated in Fig. 3E is performed using FFR-based stick–slip locomotion. As shown in Fig. 3E-i, due to the applied $B_{D}$, the FFR is initially located away from the coupled robots. An SMF is then applied to reposition the FFR at the end of either robot located opposite to the intended direction of locomotion, in this case R2, after which the FFR is oscillated along the direction of locomotion. The SMF required to position the FFR at a distance *l_b_*/2 (with *l_b_* denoting the robot’s body length) from the centroid of R2 along the direction opposite to $\vec{n}$ is calculated as:

$$\mathrm{SMF}=-G\left(p_{r2}-\frac{l_{b}}{2}\vec{n}\right)$$ (11)

While the FFR is oscillated using B_osc_, as described in Eq. (9), stick–slip locomotion is induced in both robots along $\vec{n}$. However, because the FFR is positioned adjacent to R2, the resulting magnetic interaction between the robots becomes repulsive. This repulsive interaction hinders the propulsion of R2 while promoting the forward motion of R1, resulting in a higher locomotion speed of R1 and the gradual formation of a gap between the 2 robots. Once the gap between the nearest ends of R1 and R2 reaches at least one *l_b_*, the FFR is repositioned at the midpoint between the 2 robot centroids according to:

$$\mathrm{SMF}=-G\left(\frac{p_{r1}+p_{r2}}{2}\right)$$ (12)

Then, as shown in Fig. 3E-ii, the FFR is oscillated again by applying $B_{\mathrm{osc}}$. Because the FFR is now positioned between the 2 robots, its oscillation occurs in opposite directions relative to R1 and R2, inducing stick–slip locomotion in opposite directions and thereby further increasing the gap between them.

### FFR-based selective locomotion of PMRs and speed tuning under constant RMFs

As summarized in Fig. 2, FFR-based selective control of magnetic robots relies on 3 magnetic field components: the SF, which contains the FFR; the locomotion control field, typically an RMF; and an SMF used to control the FFR position. As illustrated in Fig. 4A, during selective control, the FFR is positioned at the center of the target robot (red), after which an RMF is applied. The superposition of these fields (SF, SMF, and RMF) produces an FFR that rotates around the target robot, parallel to the RMF rotation axis and perpendicular to the robot’s thrust, thereby preserving actuation near the FFR while suppressing off-target locomotion. We first used this configuration to define the operating conditions for selective locomotion of hard-magnetic robots and analyze its performance in fluids of different kinematic viscosity *ν*. Then, we examine how controlled displacement of the FFR along the propulsion axis gives rise to the velocity-tuning mode.

**Fig. 4.**
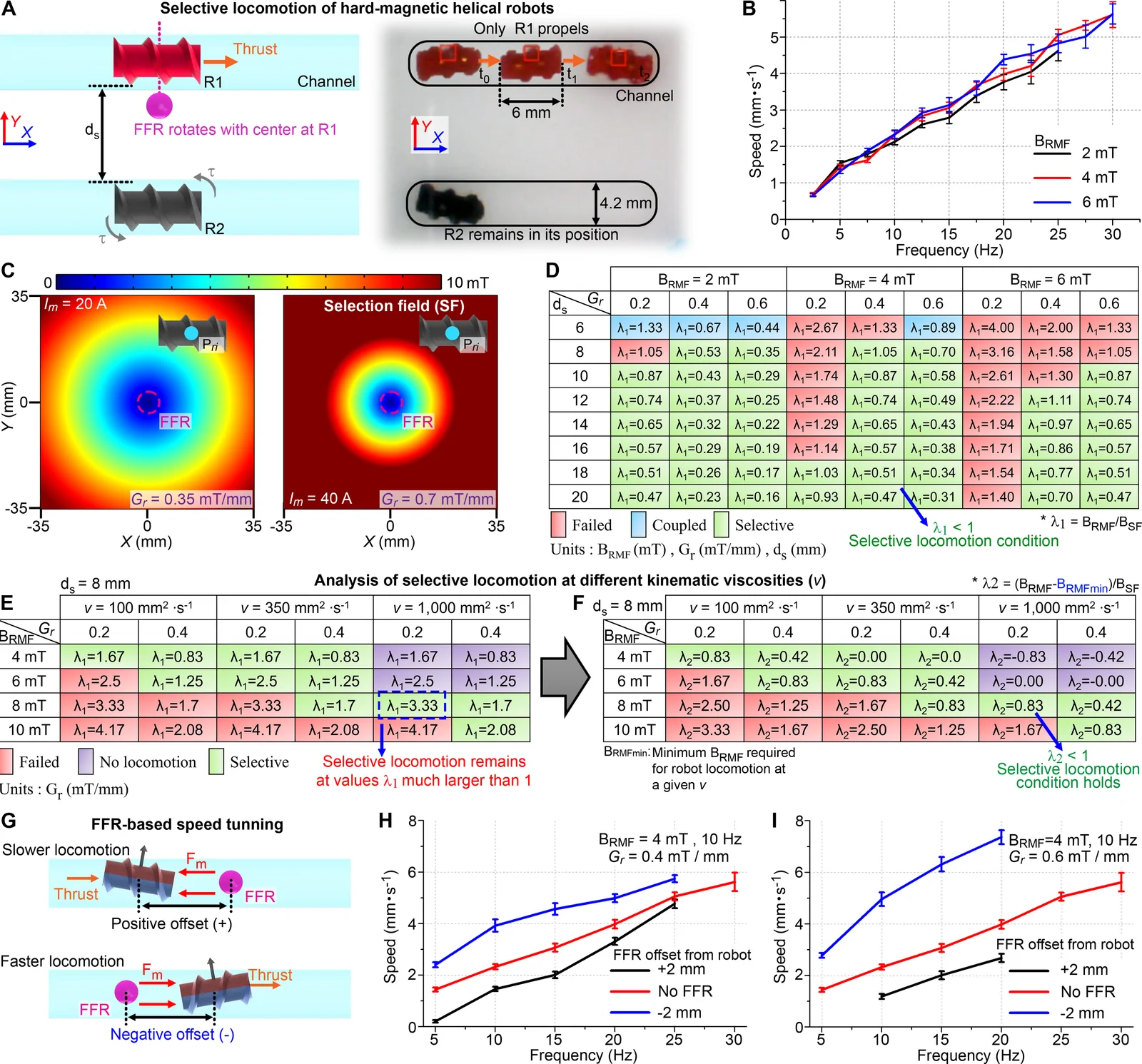
Analysis of FFR-based selective locomotion of hard-magnetic robots and FFR-based velocity tuning. (A) Selective locomotion of 2 hard-magnetic robots in parallel channels. The FFR is positioned at the target robot, enabling actuation of the selected robot (R1), while the neighboring robot (R2) remains stationary. (B) Velocity of a hard-magnetic robot (NdFeB cylindrical magnet, 4 mm in length and 1.5 mm in diameter) under RMFs of varying frequencies and magnitudes. (C) Magnetic field distributions of SFs with different radial gradients (*G_r_*). The graphs show that increasing *G_r_* increases the SF magnitude (B_SF_) at the robot center (P_ri_). (D) Magnetic field ratio λ_1_, as proposed in previous works, calculated for R2 under different *G_r_*, RMF magnitudes (B_RMF_), and inter-channel distances (*ds*). The criterion λ_1_ < 1 indicates conditions for successful selective locomotion. Green, red, and blue indicate successful selective locomotion, failed selective locomotion (both robots move), and magnetic coupling, respectively. (E and F) Tables comparing the original λ_1_ with a modified ratio λ_2_ for robots controlled in fluids with different kinematic viscosities (ν). The modified ratio incorporates the minimum RMF magnitude (B_RMFmin_) required to actuate the robot at each viscosity. (G) Schematic illustrating the principle of FFR-based velocity tuning. The FFR generates a magnetic force on the robot directed away from the FFR; therefore, offsetting the FFR relative to the robot center along or against the direction of locomotion can decrease or increase the robot velocity without modifying the RMF. (H and I) Velocity of a selectively controlled robot under a constant RMF when a small FFR offset is applied along (+2 mm) or against (−2 mm) the direction of thrust generated by robot rotation, demonstrating FFR-based velocity tuning.

Figure 4A illustrates 2 geometrically and magnetically identical PMRs placed in parallel channels separated by a distance *d_s_*. Each robot comprises an NdFeB cylindrical magnet, and to distinguish them, one robot was painted red and the other one black. The detailed specifications of these PMRs are provided in Materials and Methods. Using an FFR, the red robot is selectively driven from one end of its channel to the other, while the black robot remains stationary. Previous studies [25,26,29] have demonstrated the effectiveness of this approach for selective locomotion of different robot types. Additionally, strategies to control multiple robots by adjusting the FFR rotation diameter have been proposed [29]. However, the effect of inter-robot distance has not been previously addressed.

To develop a more comprehensive model of FFR-based locomotion, we analyzed the behavior of a single robot under varying gradients and RMFs. The robot speed was measured for RMFs of 2, 4, and 6 mT across frequencies from 2.5 to 30 Hz. As shown in Fig. 4B, the average speed increased linearly from 0.67 mm·s^−1^ at 2.5 Hz to 5.63 mm·s^−1^ at 30 Hz. The RMF magnitude (B_RMF_) had minimal influence on speed, except that operation at 2 mT was limited to frequencies below 25 Hz. Figure 4C presents the SF generated by the coil system in the *XY* plane at currents of 20 and 40 A, corresponding to radial magnetic field gradients *G_r_* of 0.35 and 0.7 mT·mm^−1^, respectively. The SF magnitude at the centroid of the ith robot, P_ri_, is denoted by B_SF_. For a given robot position relative to the FFR, B_SF_ increases with *G_r_*.

To evaluate the feasibility of selective control under different operating conditions, we use the magnetic field ratio λ_1_, as previously introduced in the literature [29]:

$$\lambda_{1}=B_{\mathrm{RMF}}/B_{\mathrm{SF}}$$ (13)

The parameter λ_1_ relates the magnitudes of the RMF and SF at the center of a given robot. During selective locomotion, the FFR is aligned with the position of the targeted robot; therefore, λ_1_ tends to infinity for this robot, indicating that the influence of B_SF_ is negligible and that the robot fully synchronizes with the applied RMF. To assess the feasibility of selective locomotion under a given set of RMF and SF conditions, λ_1_ must instead be evaluated for the nontargeted robot. Accordingly, hereafter, λ_1_ denotes the magnetic field ratio evaluated at the nontarget robot, corresponding to R2 in Fig. 4A.

The table in Fig. 4D presents λ_1_ values obtained from selective locomotion experiments conducted at 3 different *G_r_* values (0.2, 0.4, and 0.6 mT·mm^−1^) and 3 B_RMF_ values ($B_{\mathrm{RMF}}=$2, 4, and 6 mT), with the RMF frequency fixed at 10 Hz. Unless otherwise specified, the experiments using PMRs were performed in silicone oil with a *ν* of 30 mm^2^·s^−1^. The distance between channels, *d_s_*, was varied from 6 to 20 mm in 2-mm increments. Each condition is color-coded: Green indicates successful selective locomotion, red indicates failure (both robots move together), and blue indicates undesired magnetic coupling. Representative experiments are shown in Movie S1.

As observed, λ_1_ increases as *d_s_* decreases, since B_SF_ decreases as the nontarget robot approaches the FFR located at the selected robot. λ_1_ also increases with B_RMF_, and decreases with increasing *G_r_*. These results verify that selective locomotion is achievable for λ_1_ < 1. However, when *d_s_* < 6 mm, magnetic coupling occurs, preventing independent motion regardless of the value of *λ*. This operating boundary defines the regime in which selective locomotion alone is insufficient and motivates the decoupling strategy developed in a further section. Moreover, although the condition for selective locomotion, λ_1_ < 1, was satisfied in the preceding experiments, it is important to note that the PMRs could synchronize with B_RMF_ values as low as 0.5 mT at *ν* of 30 mm^2^·s^−1^. To determine whether this criterion remains valid at higher viscosities, additional experiments were conducted in silicone oils with *ν* of 100, 350, and 1,000 mm^2^·s^−1^.

Figure 4E presents the calculated λ_1_ values and the corresponding experimental outcomes for *G_r_* values of 0.2 and 0.4 mT·mm^−1^, B_RMF_ values ranging from 4 to 10 mT, and a fixed inter-channel distance *d_s_* of 8 mm. The outcomes are classified as successful selective locomotion (green), failed selective locomotion (red), and no locomotion (purple). Because $d_{s}\;$ was fixed at 8 mm, no appreciable magnetic coupling between the robots was observed. However, at higher viscosities, conditions emerged in which the robots were unable to fully synchronize their rotation with the applied RMF, corresponding to the no-locomotion regime (purple).

Notably, selective locomotion was achieved in several cases despite λ_1_ < 1, with values reaching up to 3.33, indicating that the criterion in Eq. (13) does not adequately predict selective locomotion across different fluid viscosities. This discrepancy arises because increasing the viscosity increases the B_RMF_ required to initiate robot locomotion, even in the absence of an FFR. Experimentally, the B_RMFmin_ required to propel the robots were 2, 4, and 7 mT at *ν* = 100, 350, and 1,000 mm^2^·s^−1^, respectively. To account for this viscosity-dependent actuation threshold, λ_1_ was modified as follows:

$$\lambda_{2}=\left(B_{\mathrm{RMF}}-B_{\text{RMFmin}}\right)/B_{\mathrm{SF}}$$ (14)

As shown in Eq. (14), the modified magnetic field ratio, λ_2_, incorporates B_RMFmin_ to account for the minimum RMF magnitude required to induce robot locomotion. The table in Fig. 4F presents the calculated λ_2_ values for the same experimental conditions. With the modified ratio, the criterion for selective locomotion, λ_2_ < 1, was consistently satisfied, while all cases of failed selective locomotion exhibited λ_2_ > 1. Moreover, an additional criterion emerged: When λ_2_ ≤ 0, neither robot was able to locomote. These results indicate that λ_2_ more accurately predicts the feasibility of selective locomotion across different fluid viscosities by accounting for the viscosity-dependent minimum actuation field. The previously defined λ_1_ can therefore be regarded as a special case of λ_2_ when B_RMFmin_ is negligible.

Subsequently, the average speed of the target robot was analyzed for B_RMF_ values of 2, 4, and 6 mT under different combinations of *G_r_* and *d_s_* at the initial viscosity of 30 mm^2^·s^−1^ (see Fig. S1). No clear trend was observed, with most measured speeds ranging from 2 to 3 mm·s^−1^. For reference, the robot exhibited a speed of approximately 2.3 mm·s^−1^ when actuated by the RMF alone. The observed variations are attributed to magnetic forces arising from small positional offsets between the FFR and the center of the target robot, which should ideally be negligible when the FFR is perfectly centered on the robot. This observation suggests that, beyond the basic selective-control regime, deliberately displacing the FFR along the propulsion axis could generate magnetic forces that either assist or oppose the robot’s thrust, as schematically illustrated in Fig. 4G.

To investigate this effect, selective locomotion experiments were performed under similar conditions while introducing a controlled FFR offset relative to the robot center (Movie S2). Figure 4H and I presents the measured velocities of a selectively controlled robot at *G_r_* values of 0.4 and 0.6 mT·mm^−1^, respectively, with *d_s_* = 10 mm and B_RMF_ = 4 mT, while the RMF frequency was varied from 5 to 30 Hz. Corresponding results for *G_r_* = 0.2 mT·mm^−1^ are provided in Fig. S2. For each condition, the FFR was displaced by +2 mm (positive offset) or −2 mm (negative offset) from the robot center along the thrust direction, aligned with the *X* axis. As illustrated in Fig. 4G, a +2 mm FFR offset generates a magnetic force opposing the robot’s thrust, whereas a −2 mm offset generates a magnetic force in the same direction as the thrust.

When *G_r_* was 0.2 mT·mm^−1^, the robot’s speed was nearly identical to that observed under an RMF alone, indicating that no appreciable magnetic force was exerted on the target robot. However, as the gradient increased, the robot experienced an increasing magnetic force that either hindered or enhanced its velocity depending on the FFR offset. For example, at 15 Hz, the robot’s velocity increased from 3.1 mm·s^−1^ to 4.6 and 6.3 mm·s^−1^ for *G_r_* values of 0.4 and 0.6 mT·mm^−1^, respectively, when the FFR offset was −2 mm. In contrast, at the same frequency, the velocity decreased to approximately 2.0 mm·s^−1^ for both gradient values when the offset was +2 mm. These results establish the velocity-tuning mode and show that FFR positioning can regulate robot dynamics in addition to selective locomotion.

### Stick–slip locomotion mechanism using an oscillating FFR

Traditionally, locomotion within MPI systems has been achieved by pushing or dragging magnetic robots—particularly MNPs or magnetic fluids—using the magnetic gradient forces generated around the FFR [37–39], which can impose substantial constraints on robot design and potential applications. Moreover, extending this force-based approach to the independent selective control of multiple discrete robots is challenging because the SF gradient acts throughout the workspace. Consequently, repositioning the FFR to address one robot simultaneously modifies the magnitude and/or direction of the magnetic forces acting on the remaining robots, making it difficult to immobilize nontarget robots while translating a selected robot using the same gradient field. Additional independently controlled field sources could, in principle, provide greater control over the local force distribution, but would increase the complexity of the electromagnetic system and may scale unfavorably with the number of independently controlled robots. More recent studies [25,29], including this work, have demonstrated that torque-based locomotion is also feasible within MPI systems. However, these approaches have thus far relied on RMF-driven actuation, with the FFR maintained at the center of the target robot during selective locomotion.

Here, we propose an oscillating-FFR strategy that deliberately displaces the FFR from the robot center to generate alternating magnetic torques, inducing directional stick–slip locomotion along the channel walls and thereby expanding the range of FFR-based actuation modes. As illustrated in Fig. 3A, this mechanism is designed for tubular environments, such as microchannels and blood vessels. While the control equations for locomotion along an arbitrary direction in 3D space are provided in an earlier section, a simplified 2D case is presented in Fig. 5A and B. In the first control sequence, the robot is confined within a channel extending along the *X* axis, with the intended propulsion direction along *+X*. The time-lapse images in Fig. 5A-i to A-iii show the evolution of the magnetic field distribution in the *XZ* plane as the FFR oscillates from a position directly above the robot center toward −*X* direction, parallel to the channel axis (Movie S3).

**Fig. 5.**
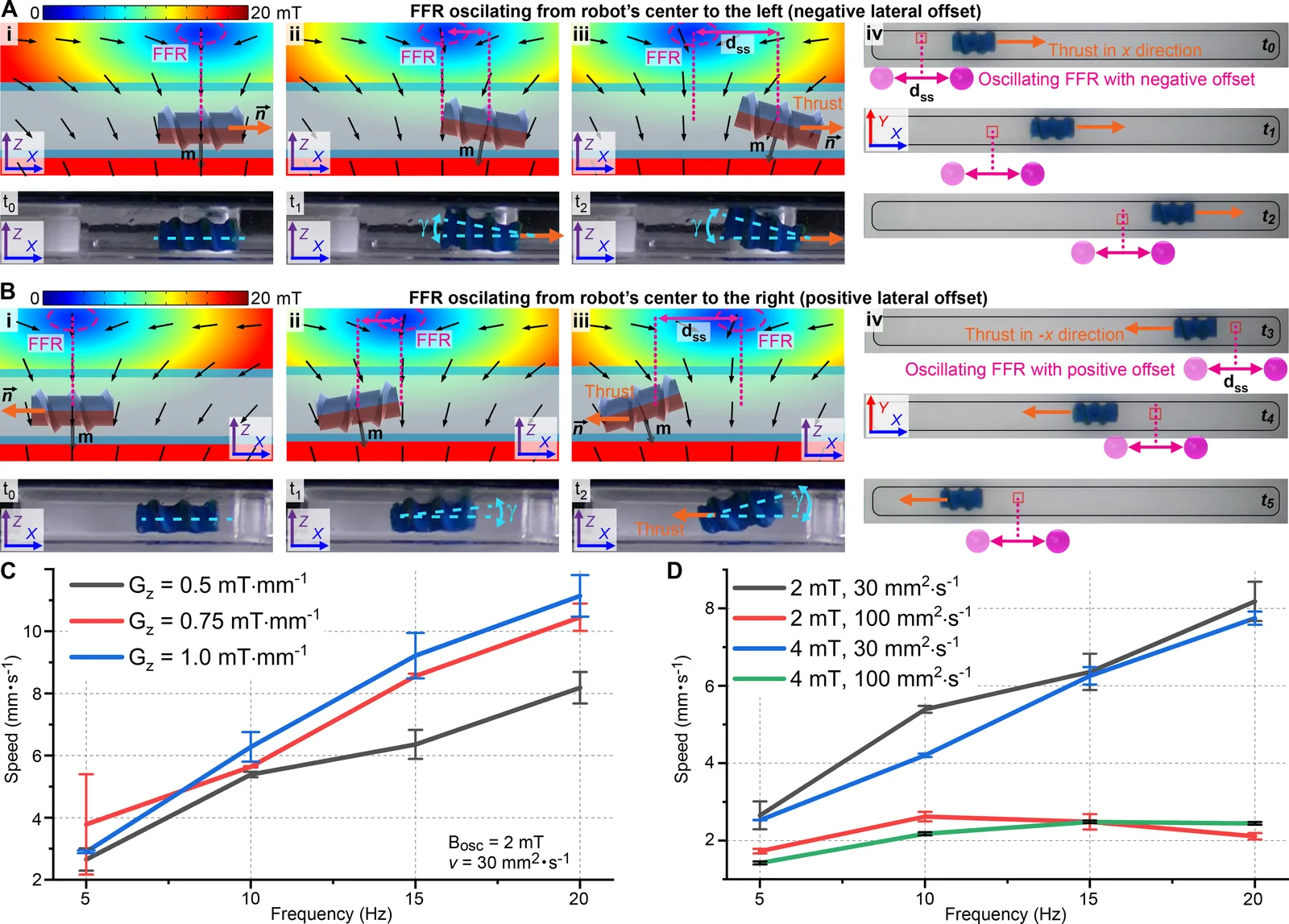
Stick–slip locomotion induced by an oscillating FFR. (A) Time-lapse images (i to iii) and corresponding magnetic field distributions showing the FFR oscillating from the robot’s magnetization axis, initially aligned with the *Z* axis, toward the −*X* direction, thereby inducing stick–slip propulsion in the opposite (*X*) direction. (iv) Time-lapse images showing the resulting robot displacement along the *X* direction, viewed in the XY plane. (B) Reversal of stick–slip locomotion by reversing the FFR oscillation direction. (i to iii) Time-lapse images showing the locomotion mechanism in the *XZ* plane, and (iv) the resulting robot displacement along the −*X* direction, viewed in the *XY* plane. (C) Measured robot speed under stick–slip locomotion at different axial gradients (G_z_) and B_osc_ frequencies in silicone oil with a kinematic viscosity (*ν*) of 30 mm^2^·s^−1^. (D) Measured robot speed at different B_osc_ magnitudes and frequencies in silicone oils with *ν* of 30 and 100 mm^2^·s^−1^.

As the FFR is displaced, the resulting local magnetic field generates a torque that rotates the robot, causing its left end to rise toward the upper channel wall and forming an angle *γ* with the channel axis. The angle *γ* increases with increasing FFR displacement. Figure 5A-iv shows the resulting displacement of the robot from left to right along the channel. During locomotion, the FFR continuously follows the robot while periodically oscillating between its initial position above the robot center and a position displaced by a constant distance $d_{\mathrm{ss}}=-r\text{tan}\left(\gamma \right)\vec{n}$ along the −*X* direction. Conversely, as shown in Fig. 5B, reversing the direction of FFR oscillation reverses the direction of robot locomotion.

Experimentally, stick–slip propulsion was observed only when the elevated end of the robot reached the opposite channel wall. We therefore define the wall-contact angle, *γ_c_*, as the rotation angle at which the elevated end of the robot first contacts the opposite channel wall. This angle represents the minimum field-induced rotation required to establish the wall-contact condition necessary for stick–slip locomotion and is geometrically determined by the channel and robot dimensions as:

$$\gamma_{c}={\text{sin}}^{-1}\left(\frac{w_{c}}{\sqrt{{l_{b}}^{2}+4{r_{b}}^{2}}}\right)-{\text{tan}}^{-1}\left(\frac{2r_{b}}{l_{b}}\right)$$ (15)

where *w_c_* and *r_b_* denote the channel width and robot radius, respectively. During the experiments, B_SF_ was set to 5 mT to ensure alignment of the robot magnetic moment with the local magnetic field at *ν* of 30 mm^2^·s^−1^. The initial FFR position and the amplitude of B_osc_ were then determined using Eqs. (7) to (9) together with the minimum rotation angle obtained from Eq. (14). Considering the dimensions of our robots, and the channel width of 4.5 mm, *γ_c_* is about 9°.

After defining the basic control parameters for stick–slip locomotion, we evaluated the influence of the SF gradient by controlling the robot at 3 axial gradients, G_z_ = 0.5, 0.7, and 1.0 mT·mm^−1^, while varying the oscillation frequency from 5 to 20 Hz at a constant B_osc_ = 2 mT. Under these conditions, the theoretical angle *γ* was 21.8°, well above the calculated *γ_c_*. Therefore, the actual angular excursion of the robot was geometrically constrained by contact with the channel walls. As shown in Fig. 5C, the robot speed increased approximately linearly with frequency at G_z_ = 0.7 and 1.0 mT·mm^−1^, showing similar behavior in both cases. The corresponding speeds increased from 3.78 and 2.90 mm·s^−1^ at 5 Hz to 10.45 and 11.14 mm·s^−1^ at 20 Hz, respectively.

At G_z_ = 0.5 mT·mm^−1^, the speed also increased with frequency but exhibited a less linear trend, reaching a maximum of 8.18 mm·s^−1^ at 20 Hz. The similar velocities obtained at G_z_ = 0.7 and 1.0 mT·mm^−1^ suggest that, above a sufficient gradient, further increases in G_z_ have a limited influence on the speed of stick–slip locomotion under the conditions investigated. In contrast, the lower and less linear velocities observed at G_z_ = 0.5 mT·mm^−1^ may be associated with its relatively low lateral gradient (G_r_ = 0.25 mT·mm^−1^), which, together with position-tracking errors, may reduce the accuracy and stability of FFR positioning.

Subsequently, we investigated the effects of B_osc_ and *ν* on robot locomotion using B_osc_ magnitudes of 2 and 4 mT in silicone oils with *ν* of 30 and 100 mm^2^·s^−1^, as shown in Fig. 5D. Although increasing B_osc_ from 2 to 4 mT increases the theoretical field-induced rotation angle, both values produce angles exceeding *γ_c_*. Consequently, the actual angular excursion of the robot is limited by the channel geometry, and increasing B_osc_ does not further increase the rotation angle once wall contact is established. At *ν* = 30 mm^2^·s^−1^, the robot exhibited similar speeds for both B_osc_ values, with measured speeds of 2.65 and 2.53 mm·s^−1^ at 5 Hz for 2 and 4 mT, respectively. These speeds increased approximately linearly with frequency, reaching 8.18 and 7.70 mm·s^−1^ at 20 Hz.

However, increasing the viscosity to *ν* = 100 mm^2^·s^−1^ substantially reduced the measured speeds. At 5 Hz, the speeds were 1.73 and 1.42 mm·s^−1^ for B_osc_ = 2 and 4 mT, respectively. For B_osc_ = 2 mT, the speed peaked at 2.62 mm·s^−1^ at 10 Hz, whereas for B_osc_ = 4 mT, the maximum speed of 2.48 mm·s^−1^ was reached at 15 Hz. Although the larger B_osc_ did not substantially increase the locomotion speed, it enabled effective stick–slip locomotion to be maintained at higher frequencies under increased viscous resistance, likely due to the greater available magnetic torque. These results suggest that, once B_osc_ is sufficient to induce the required *γ_c_*, further increases in its magnitude have a limited effect on locomotion speed. Instead, fluid viscosity plays a substantial role in determining both the achievable speed and the maximum frequency at which effective stick–slip locomotion can be maintained. Experiments at higher B_osc_ magnitudes could further evaluate this behavior; however, such fields are beyond the operating range of the current electromagnetic system for the applied G_z_ and working-space dimensions.

### FFR-based separation mechanism for magnetically coupled robots

We next experimentally evaluated the proposed decoupling strategy for restoring independent control following undesired magnetic coupling between neighboring PMRs. This limitation becomes particularly important in confined environments, where small inter-robot distances can generate strong magnetic interactions, causing robots in adjacent channels to attract and become effectively coupled across thin separating walls. Once coupled, conventional selective actuation becomes ineffective because the robots no longer respond independently to the applied magnetic fields. Here, we exploit the physical confinement provided by the channel walls to overcome this limitation. Specifically, the proposed strategy combines controlled magnetic reorientation with oscillating-FFR actuation to reduce the attractive interaction, generate differential robot displacement, and progressively restore independent control.

During selective locomotion, a controlled robot may approach another robot traveling through a neighboring channel. When the distance between their centroids, *d_c_*, becomes sufficiently small, the mutual magnetic interaction generates torques that tend to align their magnetic moments, together with an attractive force F_m_ that draws the robots toward each other. Consequently, the robots become magnetically coupled across the thin channel wall, as schematically illustrated in Fig. 3B. In this state, independent actuation is strongly hindered and conventional FFR-based selective control can no longer actuate the robots. In an unconstrained environment, strongly coupled PMRs can rotate as a single body in response to an externally applied field. In the present configuration, however, the intervening channel wall prevents direct contact and constrains their collective motion, allowing their individual orientations to be manipulated to reduce—and subsequently reverse—the attractive magnetic interaction.

The resulting separation procedure is summarized in Fig. 6A. First, a uniform decoupling field (B_D_) is applied to reorient the coupled robots and reduce their mutual attractive interaction. An FFR is then introduced near one of the robots under conditions that produce a repulsive interaction between them. Oscillation of the FFR subsequently induces the stick–slip mechanism described in the previous section, causing both robots to move in the same direction but at different speeds because the repulsive interaction hinders the locomotion of the robot adjacent to the FFR. This differential locomotion progressively generates a gap between the robots. Once the gap reaches approximately one robot body length, the oscillating FFR is repositioned between the robots, inducing stick–slip locomotion in opposite directions and further increasing their separation until independent control is restored.

**Fig. 6.**
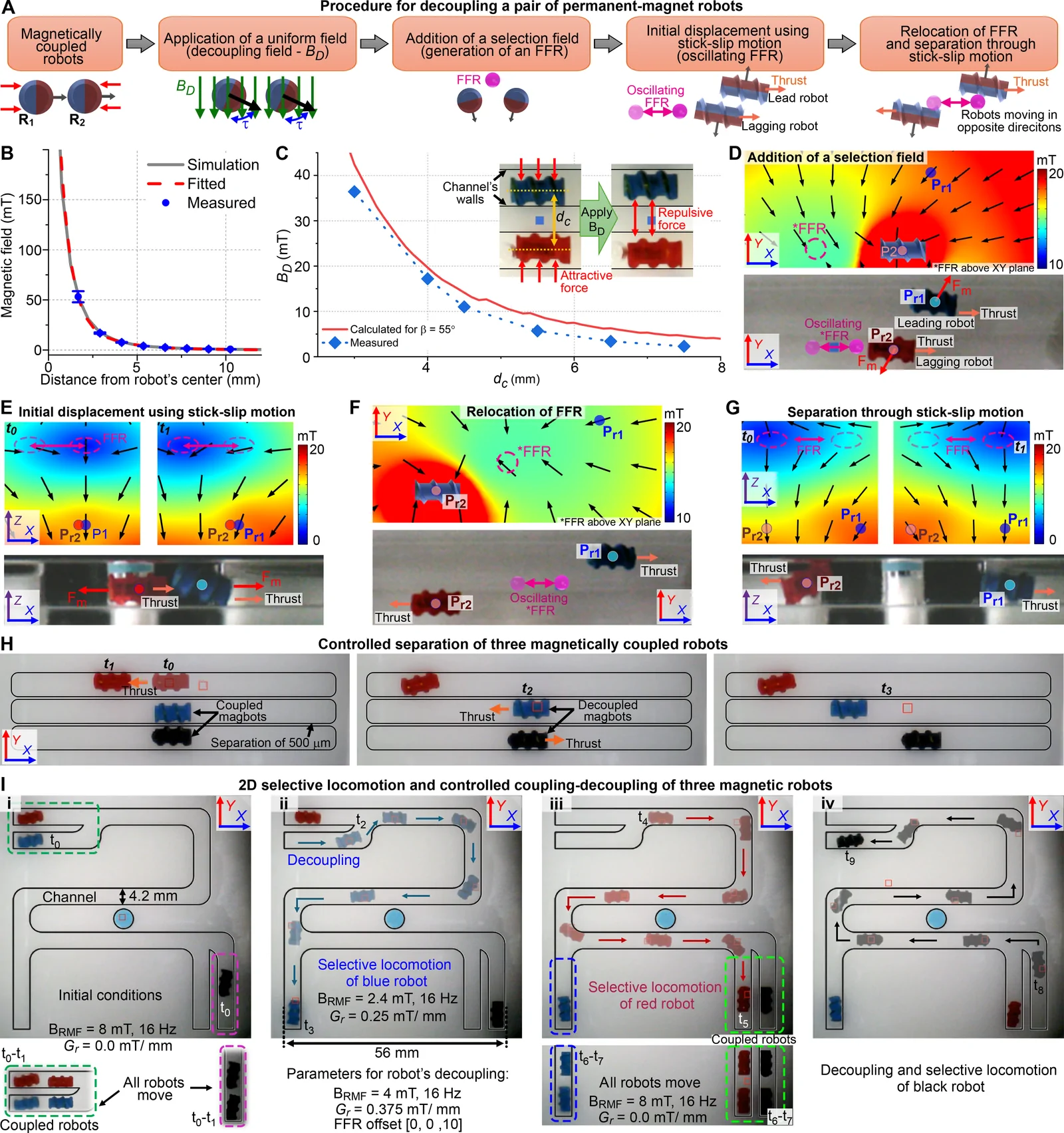
Evaluation of FFR-based separation of channel-confined magnetic robots. (A) Algorithm for the separation of 2 magnetically coupled robots. (B) Comparison of the FEM-simulated, fitted, and experimentally measured magnetic field along the magnetization axis of a magnetic robot. (C) Comparison between the calculated and experimentally measured decoupling fields (B_D_) required to reorient the coupled robots to β = 55°. The inset shows 2 initially coupled robots separating following the application of B_D_. (D) Schematic and magnetic field distribution showing the subsequent positioning of the FFR along the longitudinal axis of R2 (centroid P_r2_) while B_D_ is applied. Under this configuration, the magnetic interaction between the robots becomes repulsive. (E) Initial oscillation of the FFR from the vicinity of R2 toward the −*X* direction induces stick–slip locomotion of both robots along the +*X* direction. However, the mutual repulsive interaction hinders the locomotion of R2 relative to R1, resulting in the formation of a gap between the robots. (F) Once the gap between the robots reaches at least one robot body length, the FFR is repositioned midway between the 2 robots. (G) The FFR is subsequently oscillated along the *X* axis. Because the FFR oscillation occurs in opposite directions relative to the 2 robots, stick–slip locomotion is induced in opposite directions, further increasing their separation. (H) Controlled coupling and subsequent separation of 3 magnetic robots confined within parallel channels separated by 500-μm-thick walls, demonstrating the applicability of the strategy beyond 2-robot configurations. (I) Restoration of selective control during navigation in a 2D channel, where repeated coupling events occur as 3 robots traverse regions with small inter-robot spacing.

As mentioned, the procedure starts by applying a B_D_ perpendicular to the orientation of the coupled robots. To estimate this field, it is necessary to know the magnetic field distribution of the robot. While it is difficult to obtain through just measurements, it can be estimated from finite element method (FEM) simulations and then the data can be used to mathematically fit it and estimate the magnetic field of a robot, for instance, R2, at any given point [e.g., the centroid of R1 (pr1)]. We performed FEM simulations in COMSOL to model a permanent magnet with the say geometry as one of our robots and adjust its magnetization value to match measurements that were performed along the axis of magnetization of our robots. Figure 6B shows the FEM-simulated field, the fitted field, and the measured field along the axis of robot, displaying a strong congruence between these values.

Subsequently, the required decoupling field, B_D_, was calculated using the iterative procedure detailed previously. Briefly, the total magnetic field at each robot centroid was determined from the superposition of B_D_ and the field generated by the neighboring robot, assuming that the magnetic moment of each robot aligns with the local total field. B_D_ was progressively increased while updating the robot orientations and their corresponding mutual magnetic fields until the prescribed orientation was reached. To experimentally validate this approach, we measured the B_D_ required to transition 2 coupled robots from an attractive to a repulsive interaction at different *d_c_*, and compared these values with the calculated B_D_.

Figure 6C compares the experimentally measured B_D_ with the calculated field required to reorient both robots to β = 55°, selected slightly above the theoretical critical angle of 54.7°. Experimentally, the required B_D_ was identified as the field at which the interaction between the robots transitioned from attractive to repulsive. Measurements were performed over *d_c_* = 3 to 7.5 mm, with *d_c_* = 3 mm corresponding to directly coupled robots without a physical barrier between them and *d_c_* = 7.5 mm corresponding to an approximately 4-mm gap between their nearest edges. In the absence of confinement, directly coupled magnets would tend to rotate collectively as a single body under B_D_, preventing their relative reorientation. Therefore, for measurements at *d_c_* = 3 mm, the robots were placed within a channel that allowed individual rotational motion while mechanically restricting collective rotation of the coupled pair. This configuration enabled the robots to reorient in response to B_D_ and allowed the attraction-to-repulsion transition to be experimentally identified (Movie S4). The inset in Fig. 6C shows the robot configurations before and after application of B_D_.

As shown in Fig. 6C, the calculated B_D_ required to reach β = 55° showed reasonable agreement with the experimentally measured values and reproduced their strong dependence on *d_c_*. At *d_c_* = 3 mm, the calculated B_D_ was 42.4 mT, compared with an experimentally measured value of 36.4 mT. As *d_c_* increased, the measured B_D_ decreased sharply to 11 and 2.3 mT at *d_c_* = 4.5 and 7.5 mm, respectively, while the corresponding calculated values were 14.14 and 4.58 mT. Although the calculated values consistently exceeded the experimental measurements, both exhibited the same decreasing trend with increasing *d_c_* and were of comparable magnitude. These results indicate that the proposed calculation provides a practical estimate of the B_D_ required to overcome the attractive interaction between coupled robots. Moreover, selecting a target orientation moderately above the theoretical critical angle of 54.7° can provide an additional margin to ensure that the interaction becomes repulsive despite experimental deviations from the idealized model.

Once the attractive interaction is overcome by B_D_, the robots are physically separated using the FFR-based stick–slip mechanism (Movie S5). In the configuration shown in Fig. 6D, B_D_ is applied along the *Z* axis, perpendicular to the robots’ *XY* plane of locomotion. Because B_D_ remains applied during this stage, its superposition with the SF inherently displaces the FFR along the *Z* direction. Without modifying this *Z* coordinate, the FFR is then repositioned within the *XY* plane adjacent to one of the robots, in this case R2. Thus, B_D_ maintains the robots in the repulsive configuration, while the in-plane position of the FFR can be independently adjusted to initiate the subsequent separation motion.

As illustrated in Fig. 6E, the FFR is then oscillated from the vicinity of P_r2_ toward the −*X* direction, inducing stick–slip locomotion of both robots toward +*X*. However, because the robots magnetically repel each other, the resulting interaction acts in opposite directions on the 2 robots: It opposes the +*X* locomotion of R2 while assisting the displacement of R1. Consequently, the robots exhibit differential locomotion, and a gap progressively forms between them. Once their separation reaches at least one *l_b_*, the FFR is repositioned midway between the 2 robots (Fig. 6F). The FFR is subsequently oscillated along the *X* axis (Fig. 6G). Because the same FFR oscillation occurs in opposite directions relative to the 2 robots, it induces stick–slip propulsion in opposite directions, further increasing their separation and ultimately restoring independent control.

To further validate the proposed decoupling strategy, we performed controlled coupling and separation experiments in increasingly complex multi-robot channel environments. These experiments were designed to determine whether independent control could be restored after magnetic coupling, first in deliberately formed coupled configurations and subsequently during selective locomotion in channel networks where coupling events can occur dynamically. We first investigated whether the separation mechanism developed for 2 coupled robots could be extended to a multi-robot configuration by sequentially separating individual robots from a magnetically coupled group.

To demonstrate this capability, 3 PMRs were intentionally coupled while confined within parallel channels separated by 500-μm-thick walls (Movie S6). As shown in Fig. 6H, the upper, middle, and lower channels contained the red, blue, and black robots, respectively, which were initially aligned at the center of their respective channels and magnetically coupled across the channel walls. The separation procedure was then applied sequentially. First, the red robot was decoupled from the 3-robot configuration and displaced toward the left (t_0_–t_1_), leaving the blue and black robots magnetically coupled. The blue robot was subsequently decoupled from the black robot and likewise displaced toward the left (t_2_–t_3_), resulting in the complete separation of the 3 robots. This experiment demonstrates that the proposed mechanism can be extended beyond 2-robot configurations by sequentially decoupling individual robots from a magnetically coupled group. Furthermore, the separation direction is not intrinsically fixed by the mechanism but can be controlled through the positioning and oscillation direction of the FFR. An extended experiment demonstrating sequential separation toward the opposite direction (i.e., toward the right side of the channels) is provided in Section S3.

We then tested the FFR-based separation mechanism during the selective locomotion of 3 robots within a 2D channel containing sections where the inter-channel distance was sufficiently small (*d_s_* = 2 mm) to induce magnetic coupling, as shown in Fig. 6I (Movie S6). As illustrated in Fig. 6B-i, the upper-left region of the channel consisted of 2 parallel paths containing the red and blue robots, while the black robot was initially located in the lower-right region, where 2 channels merged into one. The red and blue robots were magnetically coupled, as demonstrated under an RMF of 8 mT at 16 Hz (t_0_–t_1_). First, the blue robot was separated and selectively guided toward the lower-left section of the channel (t_2_–t_3_, Fig. 6B-ii) using an RMF of 4 mT at 16 Hz and an SF with *G_r_* of 0.375 mT·mm^−1^. The red robot was then repositioned toward the lower-right section, where it approached the black robot and became magnetically coupled (t_4_–t_5_, Fig. 6B-iii). This coupling was evidenced by the loss of independent control, which could only be overcome by increasing the RMF to 8 mT and suppressing the SF (t_6_–t_7_). Finally, the black robot was separated and subsequently guided toward the upper-left section of the channel using selective locomotion. Thus, the decoupling strategy restores independent control during navigation in environments where coupling events would otherwise interrupt selective operation.

### FFR-based selective locomotion of low-remanence magnetic hydrogel robots

We next examined whether the selective-control framework could be extended from hard-magnetic robots to soft-magnetic hydrogel spring robots, hereinafter referred to as SMRs (Movie S7) and low-remanence magnetic robots. We first investigated selective locomotion using alginate springs containing Fe_3_O_4_ MNPs. Unlike PMRs, these SMRs exhibit superparamagnetic behavior arising from the Fe_3_O_4_ MNPs incorporated during fabrication, resulting in negligible net magnetization in the absence of an external magnetic field. Consequently, stronger RMFs are generally required to generate sufficient magnetic torque for locomotion compared with PMRs. It therefore remained unclear whether the selective-control conditions established for PMRs could be directly extended to SMRs. We investigated whether the selective-control criterion, λ_2_, remains applicable to SMRs when B_RMFmin_ is incorporated into the criterion.

Unless otherwise specified, all experiments in this section were performed in deionized water (*ν* = 1 mm^2^·s^−1^). As shown in Fig. 7A, the FFR was first centered at the target robot R1, after which an RMF was applied, causing the FFR to rotate around the target robot. Similar to the selective locomotion observed for PMRs, the time-lapse images show that only R1 translated along its channel, whereas the nontarget robot R2 remained stationary. Figure 7B shows the average locomotion speed of the Fe_3_O_4_ SMR as a function of RMF frequency for B_RMF_ values of 2, 4, and 6 mT. At 2 mT, the SMR exhibited only limited locomotion and ceased moving at frequencies above 7.5 Hz. At higher RMF magnitudes, its velocity increased approximately linearly with frequency before reaching step-out, with step-out frequencies of 12.5 and 17.5 Hz at B_RMF_ and 6 mT, respectively.

**Fig. 7.**
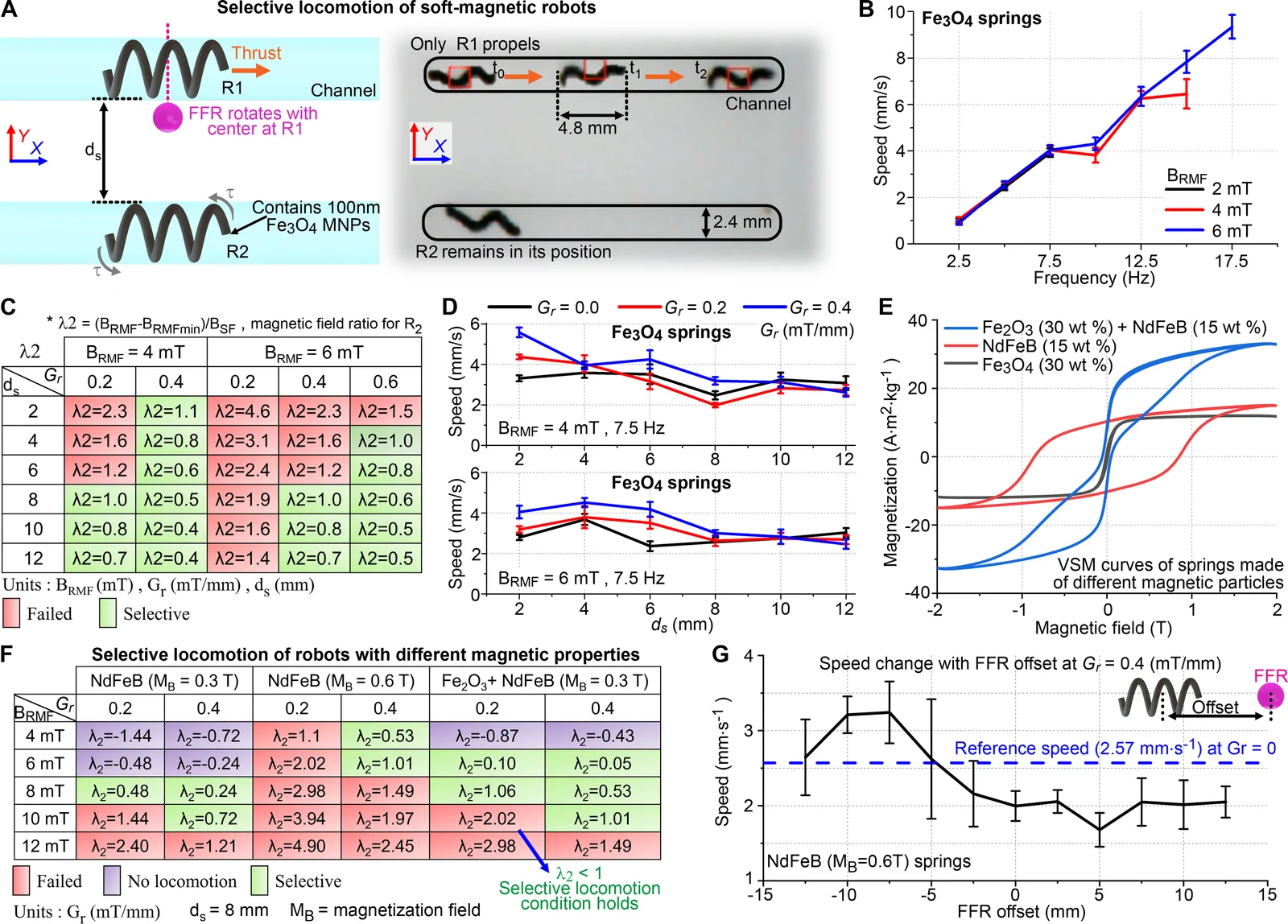
Evaluation of FFR-based selective locomotion of SMRs. (A) Selective locomotion of a SMR using the same FFR-based strategy employed for hard-magnetic robots. The FFR is positioned around the target robot (R1), enabling its actuation under the RMF, while the nontarget robot (R2) remains stationary. (B) Velocity of a soft-magnetic alginate spring containing Fe_3_O_4_ MNPs under RMFs of varying frequencies and magnitudes. (C) Comparison of successful (green) and failed (red) selective locomotion of Fe_3_O_4_ springs with the corresponding magnetic field ratio, λ_2_, for different radial gradients (G_r_), RMF magnitudes (B_RMF_), and inter-channel distances (*d_s_*). (D) Measured velocity of selectively controlled Fe_3_O_4_ springs under RMFs of varying B_RMF_ (4 and 6 mT) at a constant frequency of 7.5 Hz, for different *d_s_* and G_r_. (E) VSM measurements of 3 soft-magnetic alginate springs with different magnetic compositions: Fe_3_O_4_ MNPs (30 wt %), NdFeB particles (15 wt %), and a combination of Fe_2_O_3_ (30 wt %) and NdFeB (15 wt %). (F) Comparison of successful selective locomotion (green), failed selective locomotion (red), and no locomotion (purple) with the corresponding λ_2_ values for magnetic springs with different magnetic properties. M_B_ denotes the magnetic field at which the springs were initially magnetized. The 3 springs were analyzed at varying B_RMF_ (4 to 12 mT), varying G_r_ (0.4 and 0.6 mT·mm^−1^), and constant *d_s_* = 8 mm. (G) Influence of FFR offset on the locomotion speed of an NdFeB spring during selective control at a constant G_r_ of 0.4 mT·mm^−1^ and RMF frequency of 8 Hz.

As introduced earlier, B_RFMmin_ denotes the minimum RMF magnitude required to actuate a robot under a given set of operating conditions. Importantly, B_RFMmin_ should not be regarded solely as a viscosity-dependent parameter, but rather as an effective actuation threshold that collectively reflects the magnetic and mechanical properties of the robot and its interaction with the surrounding environment. For uniformly magnetized robots with well-defined geometries, this threshold can, in principle, be analytically estimated by balancing the available magnetic torque against the opposing hydrodynamic and mechanical torques under simplified conditions [40]. Such an approach becomes considerably less reliable for the hydrogel SMRs considered here because of their deformable and nonideal geometries, spatially distributed magnetic particles, potentially nonuniform magnetic response, and interactions with the confining channel walls. These effects are inherently coupled, are difficult to quantify independently, and may also vary among fabricated robots.

Rather than separately characterizing and analytically modeling each of these contributions, B_RFMmin_ can be readily determined through simple preliminary locomotion experiments, such as those presented in Fig. 7B, by progressively varying B_RFM_ and identifying the lowest field magnitude at which the robot begins to follow the RMF and exhibits sustained locomotion. This experimentally determined threshold inherently accounts for the combined effects of the robot’s magnetic properties, geometry, hydrodynamic resistance, confinement, and fabrication variability under the actual operating conditions. For the Fe_3_O_4_ SMRs investigated here, B_RFMmin_ was experimentally determined to be approximately 2 mT. At this field magnitude, however, the available magnetic torque was relatively low, and the robot reached step-out at only 7.5 Hz, as shown in Fig. 7B. Thus, B_RFMmin_ represents the onset threshold for RMF-driven locomotion rather than the field required to maintain synchronous actuation over the entire frequency range. This experimentally accessible parameter can therefore be incorporated into λ_2_ without requiring a detailed analytical model of each robot and its surrounding environment.

To verify the applicability of λ_2_ to SMRs, we next evaluated the selective locomotion of Fe_3_O_4_ springs under different actuation conditions and compared the experimentally observed successful and failed selective locomotion with the corresponding calculated λ_2_ values. Figure 7C summarizes the results for B_RMF_ = 4 and 6 mT, *G_r_* = 0.2 and 0.4 mT·mm^−1^, and *d_s_* ranging from 2 to 12 mm, with successful and failed selective locomotion indicated in green and red, respectively. Consistent with the behavior previously observed for PMRs, successful selective locomotion was obtained when λ_2_ < 1, whereas selective control failed when λ_2_ > 1. These results demonstrate that the λ_2_ criterion established for PMRs can also predict selective locomotion of the Fe_3_O_4_ SMRs investigated here.

We additionally examined whether *G_r_* influenced the locomotion speed of the target SMR under selective-control conditions. Similar to the behavior observed for PMRs, *G_r_* had no substantial effect on locomotion speed when the FFR was centered on the target robot. Figure 7D shows the measured velocities of selectively controlled Fe_3_O_4_ SMRs for *G_r_* = 0.0 to 0.4 mT·mm^−1^, at different *d_s_* values. Only minor variations in velocity were observed among the tested gradients. These variations were attributed primarily to small deviations in FFR positioning relative to the robot centroid and fabrication-induced variations in the geometry and magnetic properties of the hydrogel springs.

To further evaluate the generalizability of the λ_2_ criterion, we expanded the analysis beyond the superparamagnetic Fe_3_O_4_ SMRs to magnetic hydrogel spring robots exhibiting substantially different magnetic characteristics. Hereafter, we use magnetic hydrogel spring robots as a broader designation encompassing both the Fe_3_O_4_-based SMRs and hydrogel springs containing magnetic particles that exhibit appreciable remanent magnetization. Three additional sets of magnetic hydrogel springs were fabricated: springs containing 15 wt % NdFeB particles magnetized under M_B_ = 0.3 T, springs containing 15 wt % NdFeB particles magnetized under M_B_ = 0.6 T, and hybrid springs containing 30 wt % Fe_2_O_3_ MNPs and 15 wt % NdFeB particles magnetized under M_B_ = 0.3 T, where M_B_ denotes the externally applied field used to initially magnetize the springs.

Figure 7E compares the vibrating sample magnetometer (VSM) measurements of the Fe_3_O_4_, NdFeB, and Fe_2_O_3_ + NdFeB springs, revealing markedly different magnetic responses. The Fe_3_O_4_ springs exhibited superparamagnetic behavior with negligible remanence, whereas the NdFeB-containing springs exhibited pronounced hysteresis and substantially higher coercivity. The Fe_2_O_3_ + NdFeB springs exhibited the highest measured magnetization among the tested compositions, consistent with their greater overall magnetic particle content, while maintaining a lower coercivity than the NdFeB-only springs. These distinct magnetic responses provide a broader range of robot magnetic properties for evaluating whether the λ_2_ criterion can be generalized beyond a specific robot composition or magnetic behavior.

The B_RMFmin_ values of the NdFeB springs magnetized at M_B_ = 0.3 T, NdFeB springs magnetized at M_B_ = 0.6 T, and Fe_2_O_3_ + NdFeB springs were experimentally determined to be 7, 1.8, and 5.5 mT, respectively. Figure 7F summarizes the resulting locomotion behavior and corresponding calculated λ_2_ values for *G_r_* = 0.2 and 0.4 mT·mm^−1^, B_RMF_ = 4 to 12 mT, and a constant *d_s_* = 8 mm. Three distinct regimes were observed: no locomotion (purple), successful selective locomotion (green), and failed selective locomotion (red). When the applied B_RMF_ was below the corresponding B_RMFmin_, the robots exhibited no locomotion, consistent with the resulting negative λ_2_ values. Once B_RMF_ > B_RMFmin_, selective locomotion was generally successful for λ_2_ < 1, whereas λ_2_ > 1 resulted in loss of selectivity. Two experimental conditions exhibited successful selective locomotion at λ_2_ = 1.01, immediately adjacent to the theoretical boundary of the proposed criterion, whereas modestly larger values, such as λ_2_ = 1.1, resulted in failed selective locomotion. Considering experimental uncertainties in the magnetic properties, robot geometry, FFR positioning, and the empirically determined B_RMFmin_, these near-boundary observations are consistent with λ_2_ ≈ 1 representing the transition between successful and failed selective control rather than a perfectly sharp experimental threshold. Overall, the results demonstrate that λ_2_ captures the observed locomotion regimes across magnetic hydrogel spring robots with substantially different magnetic compositions and properties.

Subsequently, we investigated whether deliberately offsetting the FFR could also influence the locomotion velocity of magnetic hydrogel spring robots. For this analysis, we selected the NdFeB springs magnetized at M_B_ = 0.6 T because their higher remanent magnetization produced a more pronounced response to the SF, facilitating the evaluation of offset-induced changes in locomotion velocity. Figure 7G shows the measured velocity for FFR offsets ranging from −12.5 to 12.5 mm in 2.5-mm increments, at constant *G_r_* = 0.4 mT·mm^−1^, B_RMF_ = 5 mT, and an RMF frequency of 10 Hz. The reference velocity measured in the absence of an SF (*G_r_* = 0) was 2.57 mm·s^−1^ and is indicated by the blue line for comparison.

At larger FFR offsets beyond the range shown in Fig. 7G, approximately −15 and 15 mm, corresponding to B_SF_ values of approximately 6 mT, the robot could no longer synchronize with the applied B_RMF_. In contrast, synchronous locomotion was maintained throughout the investigated range up to FFR offsets of ±12.5 mm, corresponding to B_SF_ = 5 mT. Interestingly, under these experimental conditions, the robot remained synchronized when B_SF_ was approximately equal to B_RMF_, whereas synchronization was lost when B_SF_ exceeded B_RMF_. This observation suggests that the relative magnitudes of B_SF_ and B_RMF_ may impose an additional constraint on the range of FFR offsets that can be applied while maintaining synchronous locomotion. However, this behavior was evaluated only for the present robot and actuation conditions, and further systematic investigation across different robot magnetic properties and actuation parameters would be required to determine whether a general relationship exists.

Regarding the effect of the FFR offset on locomotion velocity, negative offsets generally increased the robot velocity, with a clear enhancement observed for offsets beyond −5 mm. However, this increase was not monotonic, and the velocity decreased again at an offset of −12.5 mm. In contrast, introducing the SF reduced the velocity from the RMF-only reference value of 2.57 mm/s to approximately 2 mm/s at an FFR offset of 0 mm. The velocity subsequently remained relatively stable at approximately 2 mm/s across most positive FFR offsets, indicating that increasing the positive offset did not produce a clear additional reduction in speed. An exception was observed at +5 mm, where a more pronounced decrease occurred. Thus, although FFR offset can modify the locomotion velocity of magnetic hydrogel spring robots, the response was asymmetric with respect to the offset direction and less systematic than that observed for rigid PMRs. This behavior may reflect the more complex interplay among the distributed magnetic response, deformability, nonideal geometry, and channel interactions of the hydrogel springs. Further investigation would therefore be required to establish a quantitative relationship between FFR offset and locomotion velocity for these robots.

### 2D selective locomotion and separation of low-remanence robots and characterization of magnetic heating

Extending FFR-based selective locomotion of PMRs to an unconstrained 2D workspace presents additional technological challenges beyond the scope of this study. In low-viscosity fluids, the strong SF-induced forces acting on PMRs can produce rapid unintended translation toward the workspace boundaries, while the absence of physical confinement allows nearby robots to approach and magnetically couple. Robust open-space control would therefore require simultaneous real-time tracking of multiple robots and closed-loop adjustment of the applied fields to compensate for SF-induced motion while maintaining sufficient inter-robot separation. Developing and validating such a multi-robot feedback-control framework constitutes a separate control problem and is therefore reserved for future work.

In contrast, magnetic hydrogel robots with low remanent magnetization experience substantially weaker SF-induced forces and inter-robot magnetic interactions, making FFR-based selective locomotion in an open workspace more readily achievable. To demonstrate this capability, 3 rolling hydrogel robots (mean diameter: 3.8 mm) containing NdFeB particles and magnetized under M_B_ = 0.3 T were placed in a square container filled with deionized water, providing a 40 mm × 40 mm working area (Fig. 8A). The robots, denoted R1 to R3, were selectively actuated using a constant B_RMF_ = 3 mT at 0.3 Hz and G_r_ = 0.35 mT·mm^−1^ (Movie S8).

**Fig. 8.**
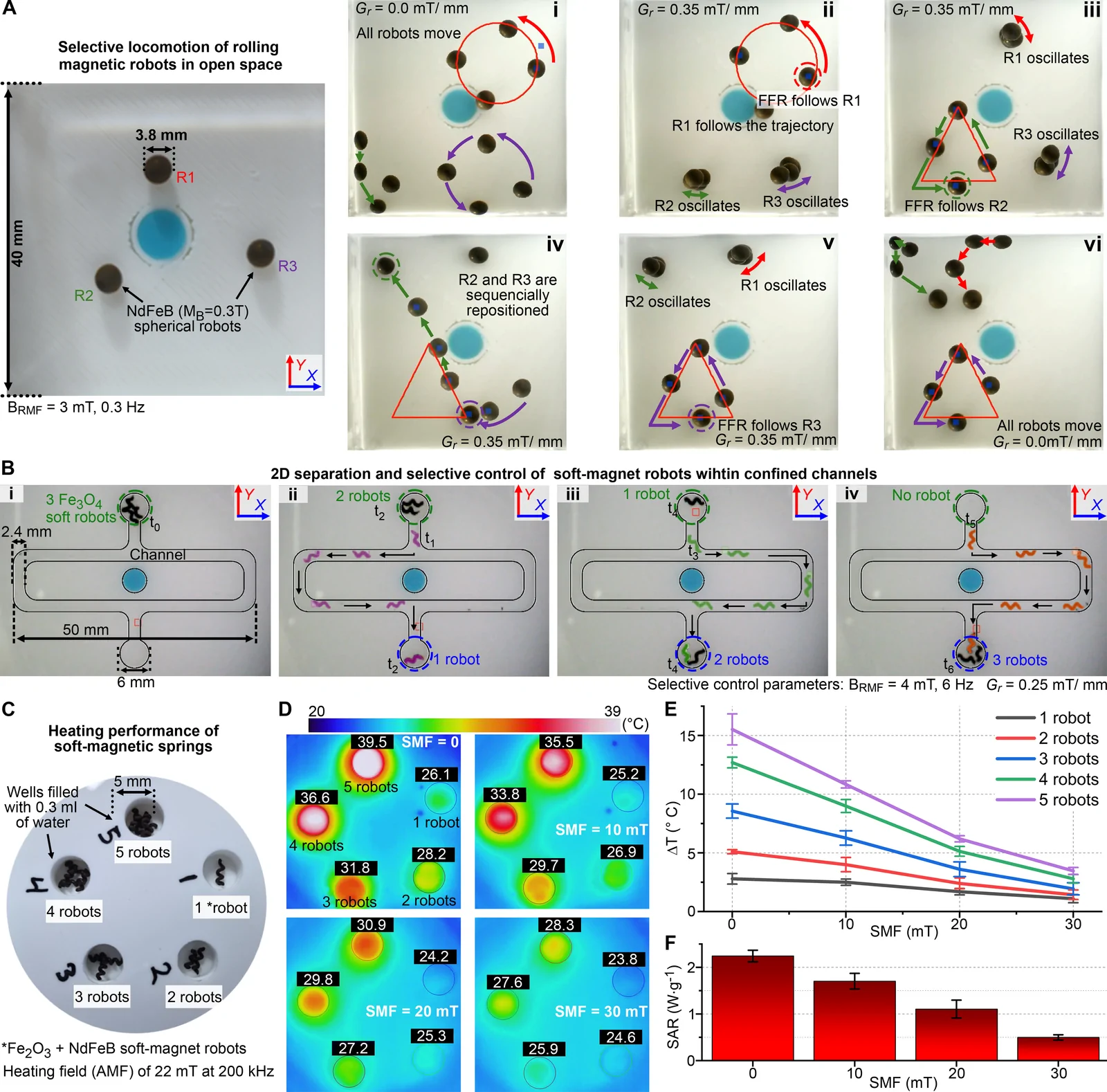
Two-dimensional FFR-based selective locomotion of magnetic hydrogel robots and magnetically controlled heating performance. (A) Selective locomotion of rolling NdFeB hydrogel robots, magnetized under M_B_ = 0.3 T, in an open 2D workspace. (B) Selective locomotion and spatial separation of Fe_3_O_4_ SMRs within a 2D channel, demonstrating that conventional FFR-based selective locomotion is sufficient when inter-robot magnetic attraction remains weak. (C) Acrylic container comprising 5 wells containing increasing numbers of Fe_2_O_3_ + NdFeB hydrogel robots, from 1 to 5 robots per well. (D) Thermal images showing the temperature increase of the Fe_2_O_3_ + NdFeB robots immersed in 0.3 ml of deionized water and exposed for 200 s to an AMF of 22 mT at 200 kHz, under superimposed SMFs ranging from 0 to 30 mT. (E) Measured temperature increase (ΔT) for wells containing 1 to 5 robots under increasing SMF magnitudes from 0 to 30 mT. (F) Measured SAR of the Fe_2_O_3_ + NdFeB hydrogel springs under increasing SMF magnitudes from 0 to 30 mT.

The time-lapse images in Fig. 8A-i to A-vi compare the motion of the 3 robots under nonselective (G_r_ = 0) and FFR-based selective control. Initially, with no SF applied, R1 was actuated along a circular trajectory (red trajectory arrows; Fig. 8A-i). Because G_r_ = 0, all 3 robots responded to the RMF and attempted to follow similar trajectories until constrained by the workspace boundaries, as observed for R2 (green arrows), which collided with the container wall. When the SF was subsequently applied and the FFR centered on R1, R1 followed the same circular trajectory, while R2 and R3 remained approximately stationary, exhibiting only small oscillations (Fig. 8A-ii). The target was then changed from R1 to R2, which was selectively guided along a triangular trajectory, while R1 and R3 (purple arrows) remained near their original positions with only slight oscillations (Fig. 8A-iii).

Subsequently, R2 was repositioned toward the upper region of the workspace and R3 was moved toward the previous position of R2 (Fig. 8A-iv), allowing R3 to follow the same triangular trajectory previously traced by R2. During selective actuation of R3, the other 2 robots again remained approximately stationary (Fig. 8A-v). Finally, the SF was removed and R3 was actuated along the triangular trajectory once more. Under these nonselective conditions, all 3 robots responded to the RMF and attempted to follow similar trajectories (Fig. 8A-vi). These results demonstrate that FFR-based selective locomotion can be extended to an open 2D workspace for magnetic robots with sufficiently low remanent magnetization, for which SF-induced translational forces and mutual magnetic interactions remain sufficiently weak to avoid substantial unintended displacement.

Moreover, no appreciable inter-robot magnetic attraction was observed during these experiments, suggesting that robots with low remanent magnetization, including the Fe_3_O_4_ SMRs, may be spatially separated using conventional FFR-based selective locomotion without requiring the active decoupling strategy developed for strongly interacting PMRs. To evaluate this, we conducted an additional experiment in a 2D channel where 3 Fe_3_O_4_ SMRs were initially placed in close contact within a circular region of 6 mm diameter, as shown in Fig. 8B (Movie S9). In this configuration, the robots were not separated physically by any barrier. Due to their superparamagnetic properties, the SMRs did not exhibit strong mutual attraction and remained responsive to external control fields. As a result, they could be separated using conventional FFR-based selective locomotion, without requiring the proposed separation mechanism.

As shown in Fig. 8B-ii to B-iv, each robot was selectively guided, one at a time, from the initial circular region (green circle) to a target region located on the opposite side of the channel (purple circle). This was achieved using an RMF of 4 mT at 6 Hz and an SF with *G_r_* of 0.25 mT·mm^−1^. The time-lapse images show the sequential selective locomotion of the 3 robots, highlighted in violet, green, and orange, respectively, while the nontarget robots remained within the initial region until individually selected. These results demonstrate that the need for an active decoupling strategy depends strongly on the magnetic interaction characteristics of the robot class. Whereas PMRs with substantial remanent magnetization can require active reorientation and separation after magnetic coupling, robots exhibiting weak inter-robot magnetic attraction can be spatially separated through conventional FFR-based selective locomotion alone.

An additional functionality of magnetic hydrogel robots containing MNPs is their ability to generate heat when exposed to a high-frequency AMF. Previous studies have demonstrated that MNP heating under a uniform AMF can be modulated by superimposing an SMF, which alters their dynamic magnetic response and consequently their heat generation [31,41]. This capability is particularly relevant to the present FFR-based control framework because spatial modulation of the SMF could potentially combine robot locomotion with localized and independently regulated heating, with potential applications including magnetic hyperthermia and microscale reaction systems. As a preliminary evaluation toward the spatially controlled heating experiments presented in a subsequent section, we therefore characterized the heating performance of the Fe_2_O_3_ + NdFeB hydrogel robots. These robots were selected because the Fe_2_O_3_ MNPs exhibited higher heating performance than the Fe_3_O_4_ MNPs used in the SMRs. As shown in Fig. 8C, increasing numbers of robots, from 1 to 5, were placed in five 5-mm-diameter wells containing 0.3 ml of deionized water. The average mass of each robot was approximately 20 mg.

The robots were uniformly exposed to an AMF of approximately 22 mT at 200 kHz for 200 s. Figure 8D shows thermal images acquired after 200 s of heating under superimposed SMF magnitudes of 0, 10, 20, and 30 mT. In the absence of an SMF, increasing the number of robots produced a substantial increase in the final temperature, from 26.1 °C for a single robot to 39.5 °C for 5 robots. In contrast, increasing the SMF progressively suppressed heating across all wells. For example, for the well containing 5 robots, the final temperature decreased from 39.5 °C at 0 mT to 35.5, 30.9, and 28.3 °C at SMF magnitudes of 10, 20, and 30 mT, respectively.

Figure 8E summarizes the corresponding temperature increase (ΔT) as a function of robot number and SMF magnitude. In the absence of an SMF, ΔT increased almost proportional with the number of robots, reaching approximately 2.80, 5.10, 8.57, 12.70, and 15.52 °C for wells containing 1 to 5 robots, respectively. Despite the different robot quantities, all wells exhibited a consistent reduction in heating as the SMF magnitude increased. At 30 mT, for example, ΔT was reduced to approximately 1.10, 1.43, 1.93, 2.79, and 3.46 °C for 1 to 5 robots, respectively. Thus, while the total heat generated could be increased by increasing the amount of magnetic material, the heating response could be simultaneously suppressed through application of the SMF.

To quantify this effect independently of the amount of magnetic material, the specific absorption rate (SAR) of the robots was determined from the heating measurements (Fig. 8F). In the absence of an SMF, the SAR was 2.24 W·g^−1^. As the SMF magnitude increased to 10, 20, and 30 mT, the SAR progressively decreased to approximately 1.70, 1.10, and 0.50 W·g^−1^, respectively. Overall, these results demonstrate that the heating output of the magnetic hydrogel robots can be substantially modulated by an externally applied SMF while maintaining a constant AMF. This SMF-dependent heating response provides the basis for integrating FFR-based spatial magnetic control with localized heating, as demonstrated in the subsequent section.

### Multimodal FFR-based multi-robot control: Proof-of-concept demonstrations

Finally, we evaluated whether the FFR-based control framework could support coordinated multimodal operation in biomedically relevant environments. Rather than focusing only on selective locomotion, these experiments were designed to test whether the framework could coordinate heterogeneous magnetic robots, preserve controllability during combined locomotion and heating tasks, and operate within more complex 3D vascular geometries. We therefore performed a series of demonstrations involving clot-drilling, targeted-delivery emulation, selective magnetic heating, and selective navigation in a cerebrovascular phantom.

First, we evaluated the separation and selective control of one PMR and 2 SMRs (Movie S10). As shown in Fig. 9A-i, the channel contained an agar clot (mimicking a blood clot) and featured sections of varying width, with all 3 robots initially in contact. The experiment began by separating the robots. The PMR (R1) was then selectively controlled to drill through the clot and restore channel patency (Fig. 9A-ii). After the clot was removed, the PMR was repositioned to the central region (Fig. 9A-iii), allowing the SMRs to navigate toward their respective targets. One SMR (R2) was guided to the upper section of the channel, as shown in the time-lapse images in Fig. 9A-iv, while the other robots remained stationary. Finally, the remaining SMR (R3) was directed to the lower region (Fig. 9A-v). This experiment demonstrates coordinated task allocation among heterogeneous robots, in which distinct agents perform clot-drilling and targeted-delivery functions within the same confined environment.

**Fig. 9.**
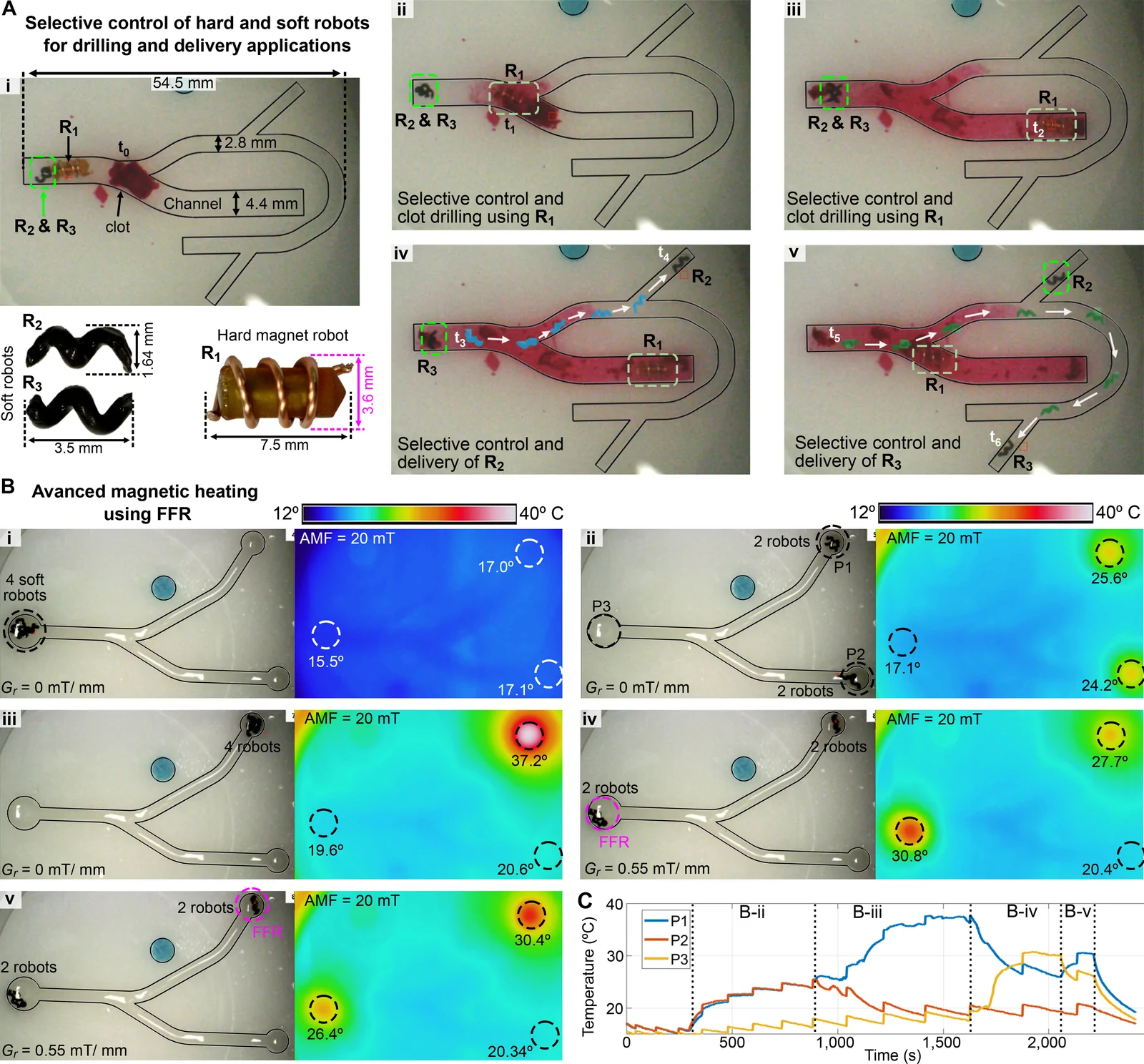
Multimodal and biomedically relevant demonstrations of the FFR-based control framework. (A) Coordinated manipulation of one PMR and 2 SMRs in an acrylic channel, demonstrating sequential clot-drilling and targeted-delivery emulation after selective separation of heterogeneous robots. (B) Selective locomotion and controlled heating of 4 SMRs, illustrating coupling of robot redistribution and thermal modulation for hyperthermia-like operation. (C) Average temperatures of the 3 target regions (P1 to P3) during the experiment in (B).

We further evaluated the FFR-based control framework as a strategy for spatially localized and tunable heating (Movie S11). The proposed approach combines 2 complementary mechanisms: controlling the number of magnetic robots accumulated within the target region to regulate its heating output, and positioning the FFR at the target region to selectively preserve heating there while suppressing heat generation by magnetic robots in nontarget regions. When the SF is applied simultaneously with the AMF, robots located near the FFR experience a negligible local B_SF_ and are therefore predominantly exposed to the AMF, allowing efficient heat generation. In contrast, robots located farther from the FFR experience progressively higher B_SF_, which acts as a local bias field and suppresses their AMF-induced heating, consistent with the SMF-dependent heating behavior demonstrated in the previous section.

To first demonstrate temperature regulation through spatial redistribution of the robots, all robots were initially concentrated within a circular region (P3) on the left side of the channel (Fig. 9B-i). The robots were subsequently distributed between 2 target regions, P1 and P2, with 2 robots positioned at each location (Fig. 9B-ii). Upon application of an AMF of 20 mT at 200 kHz, the temperatures at P1 and P2 increased from approximately 17 °C to 25.6 and 24.2 °C, respectively. The 2 robots at P2 were then transferred to P1 without interrupting the AMF, thereby increasing the local concentration of magnetic material at P1 while removing the heating source from P2. After approximately 300 s, the temperature at P1 increased to 37.2 °C, whereas that at P2 decreased to 20.6 °C (Fig. 9B-iii). This first stage demonstrates that the local heating output can be dynamically adjusted by redistributing the magnetic robots and thereby varying the amount of magnetic material present within a target region.

Next, 2 robots were moved from P1 to P3, and an FFR (*G_r_* of 0.55 mT·mm^−1^) was applied at P3 (Fig. 7B-iv). Because B_SF_ was approximately zero at the FFR, the robots at P3 remained predominantly exposed to the AMF and reached a temperature of 30.8 °C. In contrast, the robots at P1 were exposed to a B_SF_ = 27.5 mT, which acted as a local bias field and suppressed their AMF-induced heating, resulting in a lower temperature of 27.7 °C. The FFR was subsequently relocated from P3 to P1, reversing the spatial distribution of the SF and, consequently, the relative heating response of the 2 regions (Fig. 9B-v). Together, these results demonstrate 2 complementary modes of thermal regulation: Robot redistribution controls heating by varying the local amount of magnetic material, whereas FFR positioning provides spatially selective modulation of the heating response without requiring redistribution of the robots. Importantly, selective robot locomotion remained achievable throughout continuous AMF exposure, demonstrating that locomotion and thermal modulation can be integrated within the same magnetic control framework. To the best of our knowledge, this represents the first demonstration of sustained selective multi-robot control under continuous high-frequency AMF exposure, enabling multimodal operation through the combination of dynamic robot redistribution and spatially selective heating.

Next, we demonstrate the separation and selective locomotion of 2 SMRs within a 3D medical vascular phantom (Movie S12) under static-flow conditions. The phantom was filled with plasma fluid with a ν of 1.2 mm^2^/s, and no external flow was applied. Owing to the complex vascular geometry and the difficulty in automatically tracking 2 visually similar robots, their positions were manually identified by the operator from the camera views. Based on this visual feedback, the FFR position was manually adjusted to match the position of the target robot, while the desired locomotion direction was also manually specified. The corresponding magnetic fields and coil currents were subsequently calculated and generated by the control system. As illustrated in Fig. 10A, the phantom (IM Systems, Korea) replicates a section of the cerebral vasculature, including the middle cerebral artery (MCA), internal carotid artery (ICA), and posterior cerebral artery (PCA). Figure 10B presents time-lapse images of the resulting selective locomotion. Initially, both robots were in direct contact in the MCA region, without any physical barrier between them (Fig. 10B-i).

**Fig. 10.**
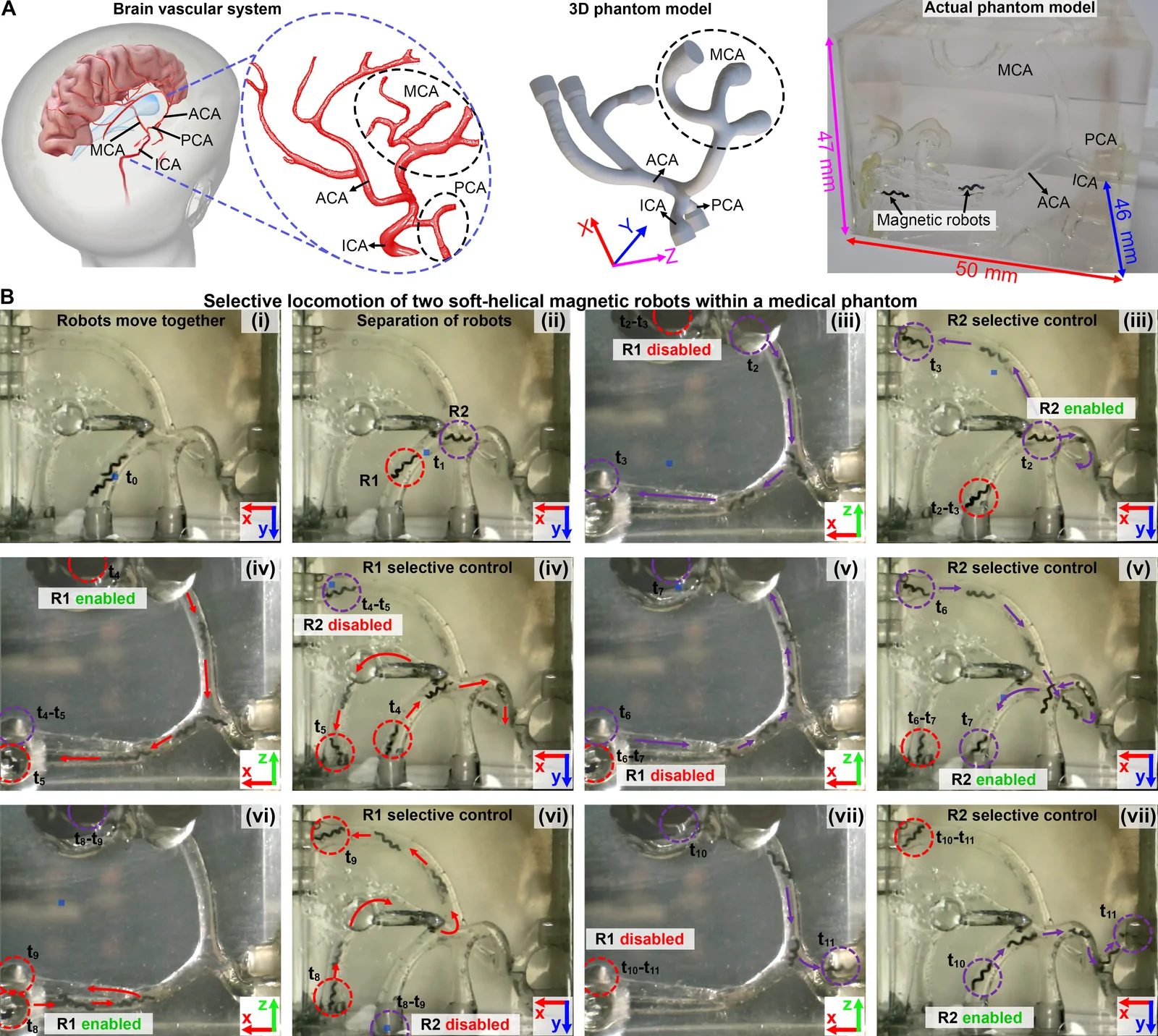
Selective multi-robot operation in a 3D cerebrovascular phantom. (A) Schematic, 3D model, and physical vascular phantom representing a section of the cerebral vasculature, containing 2 SMRs. (B) Time-lapse images showing separation and selective locomotion of the 2 SMRs through different vascular branches, demonstrating that the FFR-based control framework extends beyond planar channels to complex 3D vascular geometries.

The robots were first separated, and one robot (R2, marked in purple) was selectively guided from the MCA region into a branch diverging from the anterior cerebral artery (ACA) (Figs. 10B-ii and B-iii), while the other robot (R1, marked in red) remained in place. Subsequently, R1 was guided from the MCA into a neighboring ACA branch adjacent to R2 (Fig. 10B-iv). Then, R2 was returned to its original position in the MCA region (Fig. 10B-v), after which R1 was directed into a different vessel connected to the ACA, previously occupied by R2 (Fig. 10B-vi). Finally, R2 was repositioned from the MCA to the PCA region (Fig. 10B-vii). This experiment extended the FFR-based control framework to complex 3D vascular geometries and confirmed that selective multi-robot operation can be maintained beyond simplified planar channels.

## Discussion

This work establishes an expanded FFR-based control framework for selective locomotion, regulation of robot dynamics, deterministic decoupling, and multimodal operation of multiple magnetic robots in confined environments. A central contribution is the generalization of the selective-control criterion beyond the PMRs considered in previous FFR-based approaches. The original selective-control ratio (λ_1_) was derived under robot-specific actuation conditions and therefore could not be directly applied to robots with substantially different magnetic and mechanical responses. Here, the criterion is extended by incorporating the minimum RMF magnitude required for locomotion (B_RMFmin_) under the corresponding operating conditions. Importantly, this formulation does not imply that identical field magnitudes, gradients, or inter-robot distances are universally applicable across different robotic systems. B_RMFmin_ provides an experimentally accessible, system-specific actuation threshold that accounts for differences in magnetic response, mechanical properties, and environmental resistance. Accordingly, rather than prescribing universal field magnitudes or inter-robot distances, the framework provides a design procedure in which the robot-specific B_RMFmin_ is first experimentally determined and then used together with the local SF magnitude to evaluate whether selective actuation is expected under a given FFR configuration. The modified criterion was validated using PMRs operating in fluids of different viscosities and magnetic hydrogel robots exhibiting markedly different magnetic responses, with the predicted selective-actuation regimes remaining consistent across the tested systems. Thus, although the absolute field magnitudes, gradients, and operating conditions remain robot- and environment-dependent, the generalized criterion provides a practical basis for evaluating FFR-based selective actuation across different magnetic robotic systems.

The present framework provides sequential selective control rather than simultaneous independent actuation. At a given time, the FFR addresses a target robot and is subsequently repositioned to address another robot. Consequently, the total control time increases approximately with the number of sequentially addressed robots. However, increasing the number of robots does not require additional independently controlled magnetic sources, because the same electromagnetic system can sequentially address individual robots while maintaining nontarget robots below their actuation condition. The practical number of controllable robots is therefore determined primarily by the available workspace, tracking capability, robot dimensions, required control time, and inter-robot magnetic interactions rather than directly by the number of magnetic field sources. Similarly, the spatial addressing limit is not defined by a fixed FFR resolution, but by the selective-control criterion λ_2_. As the target and nontarget robots become closer, the SF experienced by the nontarget robot decreases, eventually preventing selective actuation. The minimum addressable distance therefore depends on G_r_, B_RMF_, and the robot-specific B_RMFmin_. For the PMRs used here in silicone oil (*ν* = 35 mm^2^/s), the criterion predicts selective addressing down to approximately 2 mm at G_r_ = 1 mT/mm and B_RMF_ = 2 mT. At such short distances, however, inter-robot magnetic interactions become substantial and impose an additional constraint not captured by the selective-actuation criterion alone. Experimentally, an edge-to-edge separation of approximately 8 mm (12.5 mm center-to-center) was sufficient to avoid meaningful coupling between the PMRs investigated here. Accordingly, no universal maximum robot number can be defined: Weakly interacting robots may be packed more densely, whereas PMRs require sufficient spacing to maintain independent addressability.

When inter-robot magnetic interactions become sufficiently strong, conventional FFR-based selective locomotion can no longer maintain independent controllability. For the strongly interacting PMRs investigated here, this regime is addressed using the dedicated decoupling field (B_D_), which reorients the coupled magnetic moments to transition their interaction from attraction toward repulsion. Oscillating-FFR-induced stick–slip locomotion subsequently provides the differential displacement required to progressively increase their physical separation. The required B_D_ was evaluated at different inter-robot distances, with the experimentally observed attraction-to-repulsion transition following the distance-dependent behavior predicted from the magnetic model. To the best of our knowledge, this represents the first experimental demonstration of the decoupling of tetherless PMRs achieved exclusively through applied magnetic fields, demonstrated here for robots confined within parallel channels separated by barriers as thin as 500 μm.

The physical barrier is an integral component of the demonstrated PMR decoupling mechanism. Rather than merely preventing direct robot contact, the barrier prevents the magnetically coupled pair from rotating collectively as a single body under B_D_, thereby forcing the relative magnetic-moment reorientation required to transition their interaction from attractive toward repulsive. In an unconstrained environment, the coupled pair may instead rotate collectively toward B_D_, and barrier-free decoupling of strongly interacting PMRs therefore remains outside the operating regime demonstrated here. Importantly, this requirement depends on interaction strength rather than representing a general limitation of FFR-based multi-robot control. Fe_3_O_4_-based SMRs and other low-remanence magnetic hydrogel robots exhibited sufficiently weak mutual interactions that initially clustered robots could be spatially separated using conventional FFR-based selective locomotion without physical barriers or a dedicated decoupling field. The appropriate FFR-based control strategy therefore depends on the competition among the applied control fields, robot-specific actuation thresholds, and inter-robot magnetic interactions.

Beyond selective addressing and decoupling, controlled manipulation of the FFR provides additional regulation of robot dynamics. Deliberately offsetting the FFR from the target robot enables its locomotion velocity to be tuned while maintaining a constant RMF, whereas oscillation of the FFR produces directional stick–slip locomotion. A geometric and magnetic wall-contact criterion defines the minimum rotation required to establish robot–wall contact and enable this locomotion mode, while experiments at different fluid viscosities, oscillation amplitudes, and frequencies characterize its dependence on the operating conditions. The oscillating-FFR strategy also serves as an enabling component of PMR decoupling by progressively increasing inter-robot separation after their magnetic interaction has been shifted toward repulsion. These capabilities illustrate that FFR manipulation can regulate not only which robot is actuated but also how the selected robot moves within the confined environment. The selective-control mechanisms investigated here are specifically developed for torque-driven magnetic robots. Although magnetic gradient forces generated around an FFR can themselves be used to transport magnetic particles, fluids, and robots, extending force-based actuation to selective control of multiple discrete robots is challenging because repositioning the FFR simultaneously modifies the force distribution acting throughout the workspace. Selective control of robots driven primarily by gradient forces would therefore require a different control formulation from the torque-based strategy investigated here.

The expanded framework also supports integration of locomotion with magnetic heating. Selective redistribution of magnetic robots changes the local quantity of magnetic material available for heat generation and consequently regulates the resulting temperature increase. In parallel, the SF provides a spatially varying static magnetic bias that can modulate the heating response under an AMF. Additional control experiments using a spatially uniform SMF showed progressive suppression of the temperature increase with increasing static-field magnitude, supporting the role of the local static magnetic bias in FFR-mediated selective heating. Thus, robot redistribution and field-mediated modulation provide complementary mechanisms for spatial regulation of the thermal response. Moreover, the SMRs remained controllable under continuous AMF exposure, suggesting that FFR-based actuation can coexist with the alternating fields required in MPI-related operation. Although simultaneous actuation and imaging remain to be demonstrated, these observations support the potential integration of FFR-based robotic control with imaging-compatible magnetic platforms in which locomotion, heating, and localization could ultimately be coordinated within a common electromagnetic architecture.

The potential biomedical applicability of this framework was explored through proof-of-concept demonstrations in simplified channels and a 3D vascular phantom with representative cerebral vascular geometry. Different magnetic robots were selectively coordinated to perform application-oriented functions including clot-removal emulation, targeted delivery, controlled heating, and selective navigation through the vascular phantom. These experiments extend the evaluation of the framework beyond basic locomotion and demonstrate the integration of selective actuation, interaction management, and functional operations in geometrically complex confined environments. Nevertheless, these demonstrations should not be interpreted as validation under physiological conditions. The experiments were performed in controlled in vitro and phantom environments without externally imposed flow or physiological pressure, and the heating experiments started from ambient temperatures and were intended to demonstrate the integration of robot redistribution and FFR-based selective heating rather than an optimized therapeutic heating protocol.

Several challenges therefore remain before translation to biomedical environments. Pulsatile flow would introduce time-dependent hydrodynamic forces and torques that could affect selective locomotion and alter the force balance associated with magnetic decoupling; consequently, the robustness of the proposed mechanisms under physiologically relevant flow and pressure conditions remains to be systematically evaluated. Although experiments at different fluid viscosities demonstrate operation under different levels of hydrodynamic resistance, they do not reproduce the time-dependent conditions associated with physiological flow. Barrier-free decoupling of strongly interacting PMRs also remains to be achieved for applications requiring operation in open or unconstrained environments, although this limitation does not directly restrict the confined operating regime investigated here. In addition, the minimum separation achievable for strongly coupled robots is constrained by the available magnetic field strength (35 mT in the present system), although this constraint may be relaxed through robot miniaturization and optimization of magnetic properties. Finally, the vascular-phantom experiments relied on optical observation and manual robot identification, while precise FFR positioning and selective control will become increasingly challenging in complex 3D environments without robust real-time feedback.

Future work should therefore focus on integrating the proposed field-shaping framework with real-time localization and autonomous control. Given the intrinsic use of an FFR, multicolor MPI represents a promising approach for simultaneously distinguishing and tracking multiple magnetic robots [42], potentially in combination with computed tomography for detailed anatomical reconstruction [43]. Such imaging capabilities could provide the feedback required for automated FFR positioning, robot selection, and adaptive control in non-optically accessible environments. Further investigation under physiological flow, pressure, and temperature conditions will also be necessary to determine the operating boundaries relevant to biomedical translation. More broadly, closed-loop systems integrating imaging, field shaping, and adaptive control may enable magnetic multi-robot systems to progress from sequential selective actuation toward intelligent task-level coordination. In summary, by integrating generalized selective actuation, regulation of robot dynamics, recovery from magnetic coupling, and multimodal functionality within a common field-shaping framework, this work expands the capabilities of FFR-based control for coordinated multi-robot operation in confined environments.

## Materials and Methods

### Fabrication of magnetic robots

The PMRs consist of a cylindrical N35 NdFeB magnet (length: 4 mm; diameter: 1.5 mm) diametrically magnetized. The cylindrical NdFeB magnet was embedded coaxially within the central region of the 3D-printed helical body, as illustrated in Fig. S4A. N35 NdFeB exhibits a remanent flux density $B_{r}$ of approximately 1.2 T, normal coercivity $H_{\mathrm{cB}}\;$ of approximately 0.9 MA/m, and intrinsic coercivity $H_{\mathrm{cJ}}$ of approximately 0.96 MA/m. The helical body of the robots was designed using 3D modeling software and fabricated via a photocurable resin 3D printer (Form 4 Basic, Formlabs). The resulting helical structure has a length of 6 mm, a diameter of 3.5 mm, and a helix pitch of 2.25 mm.

To fabricate the spring-shaped and rolling alginate magnetic robots, aqueous precursor solutions containing 3.0 wt % sodium alginate (71238, Sigma-Aldrich, USA) and the corresponding magnetic particles were prepared. Depending on the robot type, the alginate solution contained either 30 wt % Fe₃O₄ MNPs (100 nm diameter, US Research Nanomaterials, USA), 15 wt % NdFeB powder (NCZM-125-19, Nanochemazone, Canada), or a mixture of 30 wt % Fe₂O₃ MNPs (20 nm diameter, US Research Nanomaterials, USA) and 15 wt % NdFeB powder. These compositions corresponded to the Fe₃O₄, NdFeB, and Fe₂O₃ + NdFeB robots, respectively. Each mixture was homogenized using a vortex mixer for 20 min.

The resulting mixture was loaded into a 1-ml syringe and extruded through a micropipette tip into a CaCl₂ solution (C4901, Sigma-Aldrich, USA) for ionic crosslinking of the alginate. For the NdFeB rolling robots, the alginate mixture was extruded through a conventional micropipette tip. For the spring-shaped robots, the micropipette tip was cut at an angle of 20° and positioned at approximately 45° relative to the liquid surface during extrusion. This configuration enabled the formation of uniform helical (spring-like) structures. The NdFeB spring and rolling robots, as well as the Fe_2_O_3_ + NdFeB robots, were magnetized by applying a magnetizing field (M_B_) of either 0.3 T or 0.6 T along their diametric direction, depending on the experimental conditions. Photographs of all robots used in this work and measurements of their characteristic dimensions are provided in Section S4.

### Semi-automated magnetic control system and visual feedback

The complete specifications of the coil system are provided within Section S5. The electromagnetic coil system was controlled using LabVIEW. Two USB cameras (Dino-Lite, AM73115MZT) were used to simultaneously observe the *XY* and *XZ* planes of the experimental workspace, with the acquired images displayed and processed in LabVIEW. For all experiments except those performed in the 3D vascular phantom, the robot positions were automatically detected using the LabVIEW Vision Development Module. To register the camera coordinates with the magnetic coordinate system, the experimental channels incorporated a blue circular or square reference marker corresponding to the system origin. The robot positions were then determined relative to this reference point. The tracking accuracy of the robots depends on the robot type. For the SMRs, the mean position-tracking error was 2.30 mm ± 0.75 mm, primarily due to their smaller size, deformable geometry, and lower visual contrast. For the PMRs, which are larger and more readily distinguished from the background, the mean tracking error was substantially lower at 0.82 ± 0.24 mm. For the 3D vascular-phantom experiments, the same 2-camera configuration was used; however, owing to the complex vascular geometry and the difficulty in automatically distinguishing and tracking the 2 similar robots, their positions were identified manually by the operator from the simultaneous *XY* and *XZ* views.

The origin of the magnetic coordinate system was defined at the geometric center of the Maxwell coil pair. The magnetic field generated by the electromagnetic system was experimentally characterized, as presented in Section S5, confirming the spatial uniformity of the fields generated by the Helmholtz coils and the approximately linear spatial variation of the SF generated by the Maxwell coils within the experimental workspace. The nominal FFR at the system origin was further validated using a Gaussmeter (FW Bell 5170). For target G_r_ of 0.5 and 1 mT·mm^−1^, residual magnetic fields of approximately ±0.22 and ±0.37 mT, respectively, were measured at the nominal FFR, with the magnetic field increasing approximately linearly with distance from the origin. Based on the experimentally characterized field distributions, subsequent FFR positions were calculated from the superposition of the SF and uniform SMFs according to the equations described above. The magnetic-field generation uncertainty was approximately ±0.8 mT, corresponding to estimated FFR-position uncertainties of approximately 1.6 and 0.8 mm for G_r_ of 0.5 and 1 mT·mm^−1^, respectively.

The system employed a semi-automated magnetic-field control scheme in which the equations described within the manuscript were used to calculate the electrical currents required to drive the electromagnets. For RMF-based locomotion, the RMF magnitude, frequency, and orientation were specified by the operator, after which the corresponding coil currents were automatically calculated and generated. Similarly, the SF magnitude was specified by the operator, while the SMFs required to position the FFR at the detected target-robot position were automatically calculated and generated based on the tracked robot coordinates. For stick–slip locomotion, the SF magnitude, target B_SF_ at the robot position, and desired locomotion direction were specified by the operator, after which the required time-varying fields and corresponding coil currents were automatically generated. For the decoupling procedure, the inter-robot separation distance was additionally provided to the control system, which calculated the required decoupling field (B_D_) and generated it together with the fields required for the subsequent stick–slip locomotion.

### Experimental repeatability and statistical analysis

All experimental conditions were repeated at least 4 times (*n* = 4) to evaluate repeatability. For continuously varying quantitative measurements, specifically robot locomotion speed and temperature increase, the reported values represent the mean of 4 independent trials, with error bars indicating the corresponding standard deviation. Selective-locomotion and decoupling experiments were evaluated as threshold-based outcomes. For each tested condition, selective locomotion consistently resulted in the same successful or unsuccessful outcome across the repeated trials. Similarly, the decoupling field (B_D_) was experimentally determined as the threshold at which the interaction between coupled PMRs transitioned from attractive to repulsive. B_D_ was increased in increments of 0.2 mT, corresponding to the magnetic-field resolution of the experimental system. The transition was consistently observed at the same field step across the repeated trials; therefore, no variation exceeding the 0.2-mT experimental resolution was observed, and error bars are not reported for these threshold-based measurements.

## Data Availability

All data are available in the main text or the Supplementary Materials.

## Supplementary Materials

Supplementary 1 Sections S1 to S5 Figs. S1 to S6 Tables S1 to S3 Movies S1 to S12
