# Supplementary material for: Programmable Spatiotemporal Magnetic Fields for Selective Multimodal Actuation and Deterministic Decoupling of Heterogeneous Magnetic Robots
Source: Research (Wash D C). 2026 Oct 14;9:1458. doi: 10.34133/research.1458 (PMC13662276; doi:10.34133/research.1458)
Supplement: Supplementary file 1 [file research.1458.f1.zip › Supplementary Materials.docx]

***Programmable Spatiotemporal Magnetic Fields for Selective Multimodal Actuation and Deterministic Decoupling of Heterogeneous Magnetic Robots***

Supplementary Materials

**Contents**

1. **Section S1: Comparison of selective-locomotion methods**
2. **Section S2: Additional experiments for selective locomotion of PMRs**
3. **Section S3: Magnetic decoupling and separation of three PMRs**
4. **Section S4: Supplementary specifications of magnetic robots**
5. **Section S5: Specifications of the electromagnetic system**
6. **Captions of supplementary movies**

**Section S1: Comparison of selective-locomotion methods**

**Table S1.** Comparison of representative strategies for selective locomotion of multiple magnetic robots

| **Selective**  **control strategy** | **Selectivity principle** | **Robot-specific encoding required** | **Selective-control mode** | **Identical robots selectively controllable** | **Spatially defined selection** | **Spatially selective magnetic heating** | **Main advantages** | **Main limitations** |
| --- | --- | --- | --- | --- | --- | --- | --- | --- |
| Frequency-dependent selective actuation [16, 17] | Different robots exhibit distinct step-out, resonance, or frequency-dependent locomotion responses | Yes | Primarily sequential; robots are addressed by changing the actuation frequency. Simultaneous differential responses may be possible | Generally No | No | Not inherent | - Simple global-field control - Individual robots can be addressed through distinct dynamic responses | - Requires sufficiently separated frequency responses - Operating windows depend on robot properties and environment - Arbitrary simultaneous independent control is generally not available - Strong inter-robot magnetic coupling has not been addressed |
| Magnetic-property encoding [12, 13] | Different magnetization directions, magnitudes, anisotropies, or coercivities produce distinct responses to a common magnetic field | Yes | Primarily sequential or differential response depending on magnetic encoding and field protocol | Generally No | No | Not inherent | - Selectivity can be encoded directly into magnetic properties - No spatial field localization required | - Requires predetermined differences among robots - Number of distinguishable responses and independent control degrees of freedom are limited - Strong inter-robot magnetic coupling has not been addressed |
| Geometric/structural encoding [14, 15] | Different robot geometries or mechanical properties produce distinct locomotion responses under the same global field | Yes | Primarily sequential or differential response depending on robot design | Generally No | No | Not inherent | - Different locomotion modes, directions, or velocities can be generated using a common global field | - Requires heterogeneous robot designs; response depends on robot–environment interactions - Arbitrary simultaneous independent control is generally not available - Strong inter-robot magnetic coupling has not been addressed |
| Local magnetic-field actuation  [20–23] | Independently generated local fields or gradients spatially address ifferent robots | No | Potentially simultaneous independent control, depending on the number and arrangement of independently controlled field sources | Yes | Yes | Potentially, but not inherent | - Direct spatial addressing - Identical robots can potentially be controlled independently and simultaneously | - Requires additional/local field sources - Hardware complexity increases with the number of independently controlled regions/robots - Workspace may be restricted |

**Table S1.** Comparison of representative strategies for selective locomotion of multiple magnetic robots (continuation)

| **Selective**  **control strategy** | **Selectivity principle** | **Robot-specific encoding required** | **Selective-control mode** | **Identical robots selectively controllable** | **Spatially defined selection** | **Spatially selective magnetic heating** | **Main advantages** | **Main limitations** |
| --- | --- | --- | --- | --- | --- | --- | --- | --- |
| Conventional FFR-based selective locomotion [25,29,30] | SF suppresses RMF-driven rotation away from the FFR while the target robot near the FFR remains responsive to the RMF | No | Sequential spatial addressing through FFR repositioning | Yes | Yes | Yes | - Spatial selection using global fields without robot-specific encoding - Movable selection region - Compatible with MPI-type architectures and spatially selective magnetic heating | - Operating range depends on SF gradient, RMF, robot response, and spacing - Strong inter-robot magnetic coupling is not addressed - Selective locomotion primarily demonstrated under confined conditions |
| Extended FFR-based control framework (**this work**) | B_RMFmin_-based selective criterion combined with programmable FFR positioning/oscillation, speed tunning and $B_{D}$-assisted decoupling | No | Sequential spatial addressing through programmable FFR repositioning | Yes | Yes | Yes | - Selective control across different magnetic responses - FFR-based velocity regulation - Oscillating-FFR stick–slip locomotion - Active recovery from strong PMR coupling - Selective control of weakly interacting, low-remanence robots demonstrated in open 2D space - Selective heating and application-oriented functions integrated within the same field architecture | - Does not provide arbitrary simultaneous independent control - Strong-PMR decoupling currently requires geometric confinement |

**Section S2: Additional experiments for selective locomotion of PMRs**

**Fig. S1.** Measured velocity of a selectively controlled permanent- magnetic robot under RMFs with constant frequency (10 Hz) and varying rotating field magnitudes (B_RMF_), for different separation distances *ds* and radial gradients *G_r_*.

**Fig. S2.** Velocity variation of a selectively controlled robot under a constant RMF when a small FFR offset is applied along (+2 mm) or against (−2 mm) the direction of thrust generated by the robot’s rotation for a constant radial gradient (Gr) of 0.2 mT/mm.

**Section S3: Magnetic decoupling and separation of three PMRs**

**Fig. S3.** Controlled coupling and subsequent separation of three channel-confined permanent-magnet robots, separated by a wall with a thickness of 500 µm. (A) Time-lapse images showing the sequential selective locomotion of the red robot, non-selective locomotion of all three robots, and subsequent coupled locomotion of the three robots. (B–C) Sequentially controlled separation of the three coupled robots, with the target robots displaced toward the right (B) and left (C) sides of their respective channels.

**Section S4: Supplementary specifications of magnetic robots**

**Fig. S4.** Photographs of the magnetic robots used in this work. (A) Permanent-magnet robot comprising a 3D-printed UV-resin body and an NdFeB N35 permanent magnet. (B–D) Alginate spring robots comprising Fe₃O₄, NdFeB, and Fe₂O₃ + NdFeB magnetic particles, respectively. (E) Rolling alginate robot comprising NdFeB magnetic particles.

**Table S2.** Average characteristic dimensions of the robots used in this work.

| Type of robot | Length (mm) | Pitch (mm) | Diameter (mm) |
| --- | --- | --- | --- |
| Permanent magnet robot | 6.0 | 2.25 | 3.5 |
| Fe_3_O_4_ spring | 4.4 ± 0.4 | 1.9 ± 0.1 | 2.2 ± 0.1 |
| NdFeB spring | 5.3 ± 0.4 | 1.9 ± 0.2 | 2.4 ± 0.1 |
| Fe_2_O_3_+NdFeB spring | 5.28 ± 0.4 | 2.3 ± 0.2 | 2.2 ± 0.1 |
| NdFeB rolling robot | – | – | 3.8 ± 0.1 |

**Section S5: Specifications of the electromagnetic system**

Table S2 summarizes the specifications of the coils comprising the electromagnetic system. $H_{c}$, $M_{c}$, $H_{z}$, $H_{x}$, and $H_{y}$ denote the high-frequency heating coil, Maxwell coils, and Helmholtz coil pairs along the *Z*-, *X*-, and *Y*-axes, respectively. The specifications listed in the table correspond to an individual coil rather than the complete coil pair. For example, the specifications reported for $H_{x}$correspond to one of the two identical coils comprising the $H_{x}$ Helmholtz pair. The parameters N, D, R, L, B_peak_, G_Zpeak_, and P_max_ denote the number of turns, coil diameter, electrical resistance, inductance, peak magnetic flux density, peak axial magnetic-field gradient, and maximum electrical power consumption, respectively. All coils are enclosed and actively water-cooled (as shown in Fig. S2) using water maintained at 16 ± 1.5 °C to prevent overheating. Under these cooling conditions, the electromagnetic system can be operated continuously without a prescribed maximum operation time.

**Table S3.** Specifications of the coils comprising the electromagnetic system

| Coil | N | D  (cm) | R  (Ω) | L  (mH) | B_peak_  (mT) | G_z peak_  (mT mm^−1^) | P_Max_  (kW) |
| --- | --- | --- | --- | --- | --- | --- | --- |
| H_c_ | 7 | 8 | / | / | 27.6 | / | 5 |
| M_c_ | 200 | 14 | 0.47 | 5.3 | / | 2 | 1.5 |
| H_z_ | 583 | 28 | 4.47 | 139.5 | 37 | / | 1 |
| H_x_ | 430 | 44 | 3.78 | 130 | 50 | / | 3 |
| H_y_ | 602 | 60 | 7.51 | 361 | 27 | / | 3 |

**Fig. S5.** Electromagnetic system used in this work.

**Fig. S6.** Magnetic-field characterization. (A) Simulated and experimentally measured magnetic flux densities ($B_{x}$, $B_{y}$, and $B_{z}$) generated by the Helmholtz coil pairs. (B) Simulated and experimentally measured axial ($G_{z}$) and radial ($G_{r}$) magnetic-field gradients generated by the Maxwell coil pair.

Captions of supplementary movies

Movie S1. Selective locomotion of hard-magnetic helical robots.

Movie S2. FFR-based fine speed tunning of magnetic robots at constant rotating fields.

Movie S3. FFR-based slip–stick motion for magnetic robots

Movie S4. Measurement of decoupling field (B_D_) for different inter-robot distances

Movie S5. FFR-based decoupling method for the separation of magnetically coupled magnetic robots.

Movie S6. Deterministic decoupling and selective locomotion of three permanent-magnetic robots within 2D channels.

Movie S7. Selective locomotion of soft-magnetic helical robots.

Movie S8. 2D open space locomotion of hydrogel robots

Movie S9. Comparison between traditional RMF control and FFR-based selective control of soft-magnetic robots.

Movie S10. Selective separation and control of one hard-magnetic and two soft-magnetic robots within a channel, illustrating unclogging and targeted delivery capabilities.

Movie S11. Separation, selective control, and advanced thermal control of soft-magnetic robots.

Movie S12. Separation and selective locomotion of soft-magnetic robots within a 3D vascular phantom.
